# Transcriptome of GH-producing pituitary neuroendocrine tumours and models are significantly affected by somatostatin analogues

**DOI:** 10.1186/s12935-023-02863-4

**Published:** 2023-02-11

**Authors:** Rihards Saksis, Olesja Rogoza, Helvijs Niedra, Kaspars Megnis, Ilona Mandrika, Inga Balcere, Liva Steina, Janis Stukens, Austra Breiksa, Jurijs Nazarovs, Jelizaveta Sokolovska, Ilze Konrade, Raitis Peculis, Vita Rovite

**Affiliations:** 1 Latvian Biomedical Research and Study Centre, Ratsupites Str 1-k1, Riga, 1067 Latvia; 2 Riga East Clinical University Hospital, Hipokrata Str 2, Riga, 1038 Latvia; 3 Riga Stradins University, Dzirciema Str. 16, Riga, 1007 Latvia; 4 Pauls Stradins Clinical University Hospital, Pilsonu Str 13, Riga, 1002 Latvia; 5 Faculty of Medicine, University of Latvia, Raina Blvd 19, Riga, 1586 Latvia

**Keywords:** Pituitary neuroendocrine tumours, Transcriptome, Pituitary model, Somatostatin analogues

## Abstract

Pituitary neuroendocrine tumours (PitNETs) are neoplasms of the pituitary that overproduce hormones or cause unspecific symptoms due to mass effect. Growth hormone overproducing GH-producing PitNETs cause acromegaly leading to connective tissue, metabolic or oncologic disorders. The medical treatment of acromegaly is somatostatin analogues (SSA) in specific cases combined with dopamine agonists (DA), but almost half of patients display partial or full SSA resistance and potential causes of this are unknown. In this study we investigated transcriptomic landscape of GH-producing PitNETs on several levels and functional models—tumour tissue of patients with and without SSA preoperative treatment, tumour derived pituispheres and GH3 cell line incubated with SSA to study effect of medication on gene expression. MGI sequencing platform was used to sequence total RNA from PitNET tissue, pituispheres, mesenchymal stromal stem-like cells (MSC), and GH3 cell cultures, and data were analysed with Salmon—DeSeq2 pipeline. We observed that the GH-producing PitNETs have distinct changes in growth hormone related pathways related to its functional status alongside inner cell signalling, ion transport, cell adhesion and extracellular matrix characteristic patterns. In pituispheres model, treatment regimens (octreotide and cabergoline) affect specific cell proliferation (*MKI67)* and core functionality pathways (*RYR2*, *COL8A2*, H*LA-G*, *ARFGAP1*, *TGFBR2)*. In GH3 cells we observed that medication did not have transcriptomic effects similar to preoperative treatment in PitNET tissue or pituisphere model. This study highlights the importance of correct model system selection for cell transcriptomic profiling and data interpretation that could be achieved in future by incorporating NGS methods and detailed cell omics profiling in PitNET model research.

**Supplementary Information:**

The online version contains supplementary material available at 10.1186/s12935-023-02863-4.

## Background

Pituitary neuroendocrine tumours (PitNETs) are common intracranial neoplasms with variable health effects and prognosis with the prevalence of clinically significant tumours 1 per 1000 individuals [1]. Despite the fact that PitNETs are rarely metastatic, they are usually the cause of significant morbidity and mortality [2]. Although currently WHO uses expression of specific transcription factors as basis of PitNET classification (six morphologically distinct types based on transcription factors with three main cell lineages: SF1 (gonadotroph cells), PIT1 (lactotroph, somatotroph, mammosomatotroph, and thyrotroph cells), and TPIT (corticotroph cells) they can be characterised according to the type of synthesised hormone and distinct clinical symptoms [3]. Hormonally active PitNETs tend to overproduce either growth hormone (GH), prolactin (PRL), adrenocorticotropic hormone (ACTH), thyroid-stimulating hormone (TSH) and gonadotropins like luteinizing (LH) and follicle-stimulating (FSH) hormones or some combination of them [4, 5]. The majority (70–75%) of non-functioning PitNETs are gonadotroph tumours as they are immunopositive in 60% of the cases (hormone negativity in 40% of cases) for FSH and LH but do not secrete them in significant quantities [3, 6]. Current treatment strategies for patients with PitNETs are aimed to control biochemical activity and reduce tumour size while maintaining normal pituitary function or entirely remove the tumour via surgery [7]. Due to the fact that cyclic peptide somatostatin regulates pituitary hormone secretion, somatostatin binding receptors (SSTR) have become valuable therapeutic targets [8]. About 90% of GH-producing PitNETs express SSTR2 and SSTR5 which are targeted by somatostatin analogues (SSA), when intervention fails to control tumour activity dopamine agonists (DA) that target dopamine receptor 2 (D2R) are introduced to improve disease management [9]. Pegvisomant (GH receptor antagonist) can be added for acromegaly patients with only partial response to SSAs. As a result, this combination can normalize insulin-like growth factor 1 (IGF-1) levels in patients more effectively than SSA monotherapy. Since SSAs reduce excessive production of GH by PitNET, pegvisomant decreases GH actions in peripheral tissues by blocking the increased production of IGF-I in the liver [10].

Multiple studies have been devoted to investigating the PitNET transcriptome in recent years with the aim to provide a basis for PitNET classification and understanding association between tumour development, its clinical characteristics and transcriptome perturbation [11–14]. As a result, numerous genes have been identified controlling a vast array of PitNET characteristics [1]. *POU1F1* gene has shown to be involved in development of GH, PRL and TSH adenomas [12]. It is also responsible for the regulation of hormone secretion as the expression of *POU1F1* correlates with the levels of secreted PRL, TSH and GH [15]. Several coding genes (*CLDN9*, *IGFBP5, DAPK1* and *TIMP3*) and non-coding genes (*LINC00473* and *CDKN2BAS*) have been shown to be associated with the invasiveness of PitNETs [13, 14, 16]. Expression of *MUC16, MACC1 and GRHL2* has been reported to be altered in response to SSA treatment [17]. Non-coding RNA *LL21NC02-21A1.1* and protein coding gene *NOL6* have been associated with the recurrence of PitNETs [18]. The elaborate pan-genomic study of transcriptomic landscape of PitNET subgroups based on 2017 WHO transcription-factor classification [11]. The report detected that tumours with USP8 and GNAS mutations have distinct transcriptomic profiles compared to tumours without these tumour driver variants, indicating that transcriptomic studies can bring valuable information on PitNET functional aspects [11].

So far, the impact of SSA therapy on the transcriptomic landscape of PitNETs has been investigated in two studies [11, 17]. It has been demonstrated that SSA preoperative therapy downregulated Ki67 levels and upregulated *MUC1* and *CD40* expression in tumour tissue transcriptomes [11]. Additionally, expression of several tumourigenesis related factors are decreased upon SSA/DA treatment—*MUC16, MACC1*, and *GRHL2* and extracellular matrix related collagen pathways might have implications in PitNET response to medication [17]. Therefore, more studies are necessary to more comprehensively assess the impact of SSA/DA treatment on the transcriptomic landscape of PitNETs.

The most often used model cell lines used in PitNET functional studies are derived from *Rattus norvegicus* (MMQ, GH3, RC-4B) or *Mus musculus* (AtT-20) PitNET tumours, and the representation of human pathobiology of PitNETs in these cell line remains questionable. Although there are reports of human PitNET cell lines used in functional studies, widely accepted human PitNET models are not commercially available. Many authors have demonstrated free-floating sphere (pituisphere) formation obtained from the primary pituitary tumour [19–23]. According to reports, PitNETs have detectable levels of cancer stem cells (CSC), which were found in a variety of tumour types and are thought to promote tumour growth and tissue invasion, as well as resistance to therapy. CSCs are considered to be salient players in PitNET development. These cells mainly display expression of stemness markers OCT4, CD133, nestin, SOX2, and CXCR4 and demonstrate self-renewal competence [24]. We have previously demonstrated that pituispheres genetically correspond to the PitNET tissues, showing that pituispheres might be a valuable novel model system for PitNET tumourigenesis and therapy response studies [21]. Mesenchymal stromal stem-like cells (MSC) are thought to represent tumour microenvironment [25]. It has been recorded that MSCs play an essential role in tumour formation and progression via causing epithelial-mesenchymal transition (EMT). This cell type is adherent in standard culture and express surface markers CD73, CD90, and CD105 [21, 25]. We have previously demonstrated that PitNET derived MSCs do not contain somatic variants linked to the tumour [21, 26].

In this study we aimed to characterise transcriptomic patterns of GH-producing PitNETs specifically assessing impact of SSA treatment effects on gene expression on several levels and functional models of GH-producing PitNETs: tumour tissue of patients with and without SSA preoperative treatment, tumour derived pituisphere model and classically used GH3 cell lines treated with SSA. For the first time, we incorporate various complementary tumour models to derive a comprehensive assessment of SSA impact on GH-producing PitNET functionality.

## Materials and methods

### Study group

Tumour tissue samples were collected from 82 patients who underwent planned resection at Pauls Stradins Clinical University Hospital, Latvia from 2010 till 2021. Patients’ clinical data and medical treatment history is presented in Additional file 1: Table S1. All patients were recruited to the Latvian National biobank—Genome Database of the Latvian population [27]. Broad informed consent for biobank and project-specific consent for research involving the pituitary tumours were obtained from all patients (approved by the Central Medical Ethics Committee of Latvia protocol No. 22.03.07/A7 and 01.29.1/28/renewed prot. No. 01-29.1/5035, respectively).

PitNET tissue samples (after resection) were divided into two parts. One part was submerged in RNAlater Solution (Thermo Fisher Scientific, USA) for DNA/RNA extraction, and another part was immersed in Dulbecco’s Modified Eagle Medium (DMEM) (Thermo Fisher Scientific, USA) containing 1 × penicllin/streptomycin solution (GIBCO, USA) for cell culture development.

### Study design

**Fig. 1:**
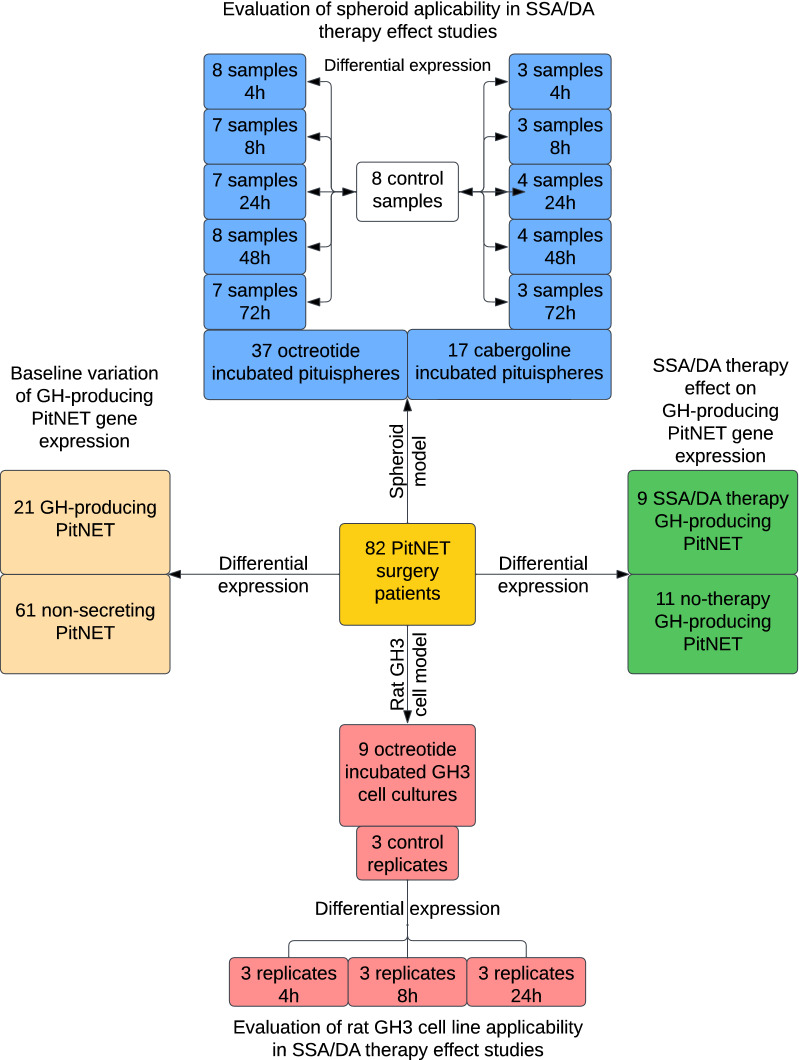
Graphic study design displaying aims, comparison groups and sample sizes

We developed a complex design to investigate various aspects of GH-producing PitNETs transcriptomics (Fig. 1). Comparison of GH-producing and non-functional PitNETs included the largest sample set with 21 and 61 samples, respectively. Within the GH-producing PitNET group there were nine patients with SSA/DA drug therapy and 11 without it. Primary derived cell cultures (MSC and pituitspheres) derived from eight PitNETs were propagated for 72 h incubated with two common PitNET drugs: octreotide and cabergoline. Finally, widely used commercial cell line GH3 was used to evaluate reproducibility of octreotide incubation related DEGs in rat model cell line. Information regarding tested sample groups and their related information is available in Additional file 2: Tables S1–S3.

### Transcriptome sequencing

Total RNA for transcriptome sequencing was extracted using AllPrep DNA/RNA/miRNA universal kit (Qiagen, Germany) according to manufacturer’s instructions from tumour tissue samples stored in RNAlater Tissue Storage Reagent (Sigma-Aldrich, USA). The concentrations of extracted RNA were measured using Qubit 2.0 with Qubit RNA HS kit (Thermo Fisher, USA). The quality of extracted RNA was assessed using Agilent 2100 Bioanalyzer (Agilent Technologies, USA). Following this the RIN and DV200 values were calculated to calculate the RNA input amounts for RNA-seq compatible library preparation. Prior to the library preparation rRNA removal was performed using MGIEasy rRNA Depletion kit (MGI, PRC). After the rRNA depletion reverse transcription, second strand synthesis, and cDNA library preparation for NGS were carried out using MGIEasy RNA Directional Library Prep Set (MGI, PRC). The paired-end libraries were sequenced on DNBSEQ-G400 platform (MGI, PRC) with the aim of 35 million reads per sample at 150 bp read length.

### Culturing of PitNET tissue material and pituisphere sequencing

Within 12 h after surgery, PitNET tissue samples were processed for propagation. Tissue material was mechanically sliced into small pieces and washed in DMEM with 1 × Antibiotic–Antimycotic solution (Thermo Fisher Scientific, USA). Enzymatic dissociation using Accutase solution (Thermo Fisher Scientific, USA) was carried out on a rotating platform for 20 min at 37 °C in a humidified environment with 5% CO2. The cells were centrifuged for 5 min at 360 × g after the incubation period to obtain cell pellets. The cell pellet was treated with a red blood cell lysis solution (154 mM NH4Cl, 10 mM KHCO3, 0.1 mM EDTA, pH 7.4) for 10 min to reduce contamination with red blood cells. To remove red blood cell debris, the sample was centrifuged, and the cell pellet was washed twice. The obtained pellet was split into two portions. To obtain PitNET tissue-derived free-floating spheres, cells were grown in DMEM-F12 (Thermo Fisher Scientific, USA), containing 1 × penicillin/streptomycin solution, 20 ng/ml epidermal growth factor (EGF) (Sigma-Aldrich, Germany), 10 ng/ml basic fibroblast growth factor (bFGF) (Sigma-Aldrich, Germany), and 1 × B27 supplement (GIBCO, USA). To obtain MSC, culture cells were grown in DMEM-F12, supplemented with 10% Fetal Bovine Serum (FBS) (Thermo Fisher Scientific, USA), 1% ITS (Corning, USA), and 100 µg/ml primocin (InvivoGen, USA) until confluent, then propagated and passaged 2–6 times. All cell culture cultivations and incubations were performed at 37 °C, 95% air, and 5% CO2. For the gene and protein expression experiments, pituispheres were grown on a 6-well plate and treated with 10 μM octreotide or 10 μM cabergoline for 4, 8, 24, 48, and 72 h.

For pituisphere transcriptome sequencing we generated and amplified 62 cDNA from tubes containing pituispheres using REPLI-G WTA single cell kit according to manufacturer’s instructions. The kit included lysis reagents and gDNA removal reagents therefore no prior RNA extraction from tubes containing pituispheres was required. cDNA was generated only from Poly A transcripts to avoid rRNA reads in sequencing. After cDNA was generated, the cDNA was amplified using multiple displacement amplification to meet the required concentrations for downstream processing. The quality and concentration of final cDNA was tested using Agilent 2100 bioanalyzer and Qubit 2.0. For fragmentation before the library preparation, we optimised a method using Covaris S220 ultrasonicator (Covaris, USA). For ultrasonication 1000 ng of cDNA was used for each sample. The following settings were used for fragmentation: target bp—300 bp, peak incident power—75 W, duty factor—20%, cycles per burst—1000, fragmentation time—45 s. Fragmented cDNA samples were visualised on Agilent 2100 bioanalyzer prior to the library preparation. 62 libraries were prepared with DNBSEQ-G400 compatible library preparation kit—MGIEasy PCR–Free DNA Library Prep Set (MGI, PRC) according to the manufacturer’s instructions. The quality and concentrations of libraries were evaluated using Agilent 2100 Bioanalyzer and Qubit 2.0. We sequenced the pituisphere paired-end libraries on DNBSEQ-G400 sequencer (MGI, PRC) with aim of 35 million reads per sample at 150 bp read length.

### GH3 cell line culturing and sequencing

Rat pituitary derived GH3 cell line was obtained from ATCC (American Type Culture Collection, USA). The GH3 cells were maintained in F12-K medium containing 15% horse serum, 2.5% FBS and 1 × penicillin/streptomycin solution. For studying the effects of PitNET drugs on gene expression, the cells were incubated for 4, 8, 24, 48, and 72 h with 10 μM octreotide. The GH3 cells were sequenced as previously described in the context of tissue material sequencing.

### Data analysis

Raw sequencing data quality control was performed using FastQC (*v0.11.9*) and MultiQC (*v1.10*) software [28]. Afterwards, the paired-end data was trimmed with fastp (*v0.23.2*) software to retain reads with average base quality of at least 20 (Phred score) and minimum read length 100 base pairs [29]. MGI/BGI sequence ID correction and overlapping read base correction were also enabled to minimise chance of incompatibility with the aligner and possibility of base level sequencing errors. Read count and quality was again inspected using the aforementioned QC software which was followed by ribosomal RNA (rRNA) removal with SortMeRNA (*v4.3.4*) before final quality and read count evaluation [30]. Reads were further quasi-mapped and quantified using Salmon (*v1.6.0*) against the GENCODE (*v38*) Homo sapiens gentrome for tissue and pituisphere samples and against Ensembl (*v105*) Rattus Norvegicus gentrome for the GH3 cell lines with GC bias, sequence level bias and positional bias correction enabled [31]. Tissue samples with quasi-mapping rate of at least 45% were selected for differential expression analysis. R software (*v4.1.1*) was employed to summarise gene level counts using the tximeta package (*v1.12.4*) [32, 33]. Differential expression analysis was performed by DESeq2 (*v1.34.0*) [34]. For tissue samples, quantified read counts were filtered by frequency setting the count threshold at 10 and the sample frequency threshold as 33% of the smallest comparison group contrast size, accordingly 3 for the SSA/DA therapy comparison and 7 for the PitNET type comparison.

Wald test was used to determine expression differences. The default independent filtering function was replaced with independent hypotheses weighing from the IHW package (*v1.22.0*) to increase statistical power by adjusting p values based on the mean expression level of each gene which was followed with multiple testing correction using the Benjamini–Hochberg adjustment [35]. Log Fold Change (L2FC) shrinkage algorithm from the apeglm package (*v1.16.0*) was used to correct L2FC values for genes with low counts and high dispersions [36]. Heatmap of gene level normalised count values was graphed by pheatmap (*v1.0.12*) package and used for visualisation of the differences between the selected differentially expressed genes (DEG) [37]. To inspect p-value variability across the full range of L2FC values, a volcano graph was drawn using the EnhancedVolcano (*v1.12.0*) package, where the threshold for p-value was < 0.05 and L2FC threshold was the same as in the shrunken results table filtering step [38]. Box plots for each DEG were constructed with the ggplot2 (*v3.3.5*) package to inspect changes in normalised count values in the possible candidate genes [39]. To gain insight in DEG involvement with signalling pathways or association with disease, enrichment was performed by using STRING-db’s (*v11.5*) online tool [40].

Most of the mentioned differential expression analysis steps above were also applied to the *Rattus norvegicus* model data of GH3 cells and in large part also to data of the pituisphere model experiments, except the Likelihood Ratio Test (LRT) was used, to perform a time-series analysis against a reduced model which allowed us to observe DEG’s with a more concurrent trend across all included time points. Subsequently a Wald test was performed between each incubation period and the unincubated controls. No L2FC shrinkage or IHW p-value correction was applied to the pituisphere LRT test or GH3 cell model results. For the incubated GH3 cell analysis, null hypothesis correction was applied to infer more accurate p-values and subsequent false discovery rate detection using the fdrtool (*v1.2.17*) [41]. Information about sample groups and related information available in Additional file 2: Tables S1–S3.

## Results

### Transcriptomic patterns of GH-producing PitNETs

As a result of PitNET tissue transcript quasi-mapping, quantification and count summarization to gene level, expression profiles of 60230 genes were obtained, which after exclusion of low expressed genes (ten counts in at least seven samples) were reduced to 22669.

**Fig. 2:**
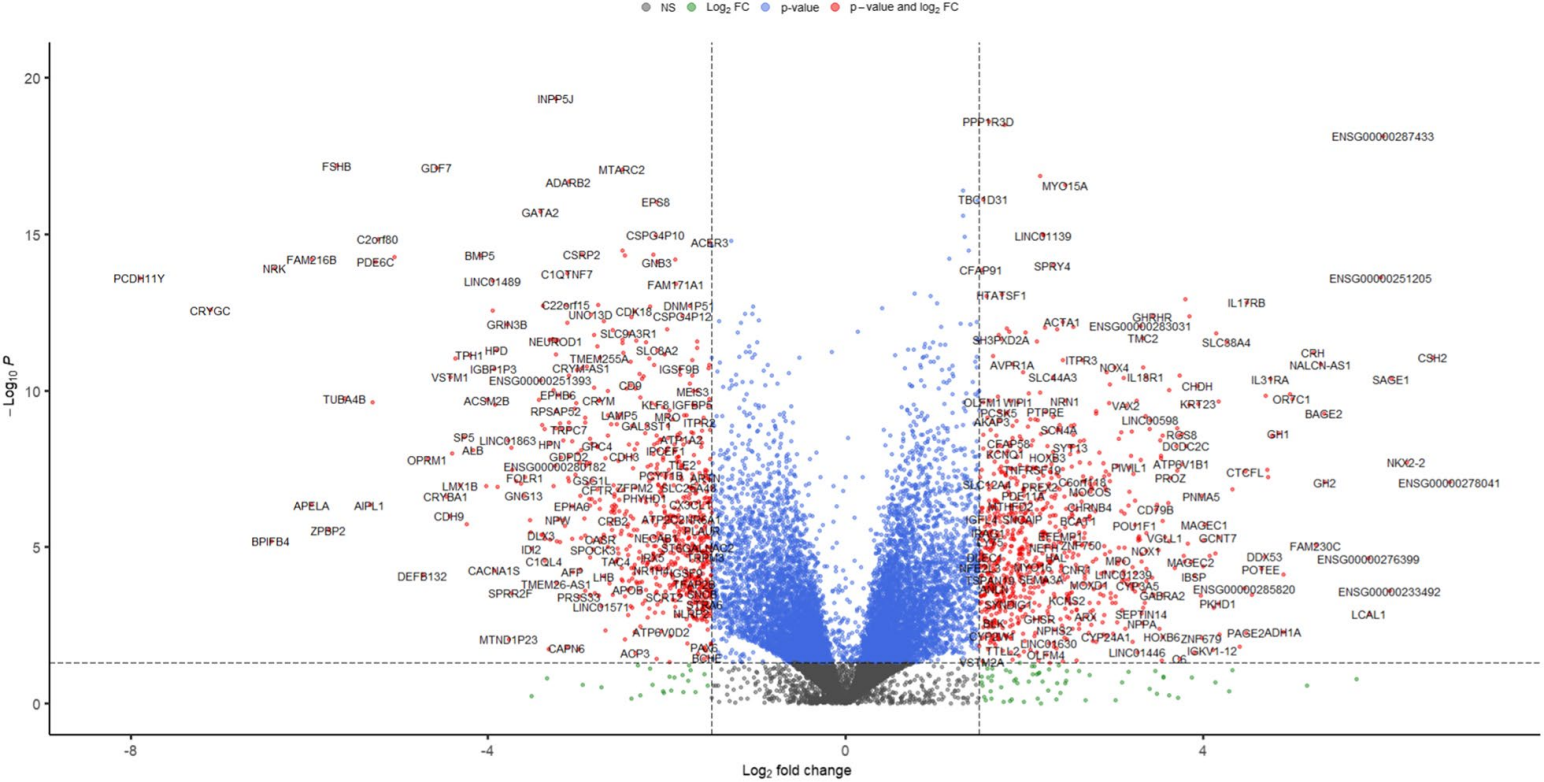
Volcano plot representing the relationship between differential expression test (Wald test) p-values and shrunken (“apeglm” algorithm) transformed L2FC values. The dashed vertical lines represent absolute L2FC threshold of 1.5 and the horizontal dashed line represents p-value threshold of 0.05. Red points denote genes passing both thresholds, blue points represent genes not passing the L2FC threshold and grey points represent points not passing any of the mentioned thresholds. See full list of differentially expressed genes in Additional file 1: Table S2

Differential expression tests between the subtypes of GH-producing PitNETs (N = 21) and non-functioning PitNET (N = 61) resulted in 1595 significant differentially expressed genes (L2FC >  ± 1.5 and p-adjusted < 0.05 thresholds). There was a similar count of upregulated (829 (54%), median L2FC = 2.1, IQR = 0.92) and downregulated DEGs (740 (46%), median L2FC = − 2.01, IQR = 0.85) in the GH-producing PitNET group (Fig. 2, Additional file 1: Table S2, Additional file 3: Fig. S1).

### Functional enrichment and protein–protein interactions in GH-producing PitNETs

**Fig. 3:**
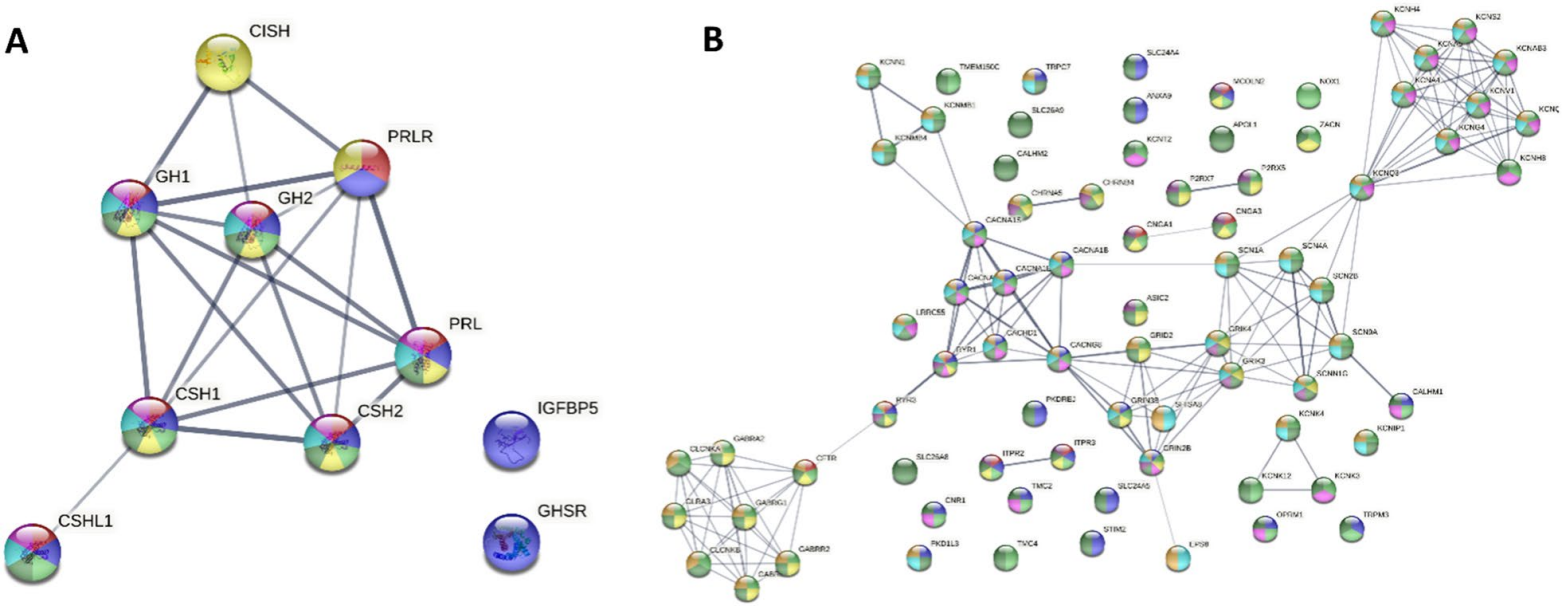
**A**. Reduced STRING-db protein interaction network for growth hormone related significantly altered enrichment terms (Additional file 1: Table S5). **B**. Reduced STRING-db protein interaction type network for RYR1 and RYR2 associated molecule transport channels (Additional file 1: Table S6). Line thickness indicates confidence level

Functional enrichment analysis of 955 (59.8%) out of 1596 genes returned 426 statistically significant results with FDR < 0.05 (Additional file 1: Table S3. and S4.). “GO Process” represented the largest number of detected enrichments with 212 entries (Additional file 3: Fig. S2). This analysis revealed significant and strong enrichment (Median strength = 1.08, IQR = 0.35) for seven pathways associated with growth hormone secretion and signalling (Additional file 1: Table S5., Fig. 3A). Strong enrichment was also detected for several different ion transport channels enriched for RYR1 and RYR3 DEG’s (Additional file 1: Table S6, Fig. 3B).

Other relevant interaction networks specific to GH-producing PitNETs in our data were related to ion channel functionality (“Potassium ion homeostasis”, “Sodium ion homeostasis”, “Voltage-gated calcium channel activity” with enrichment strength 0.8, 0.75, 0.71 respectively), calcium signalling (“Calcium channel activity”, “Calcium transport”, “Calcium channel” with enrichment strength of 0.65, 0.64, 0.64 respectively), neuronal and synaptic regulatory networks (“Regulation of postsynaptic density organisation”, “Neurexins and neuroligins”, “Synaptic membrane adhesion” with enrichment strength 0.89, 0.86, 0.74 respectively) and head structure development factors (“Pituitary gland development” and “Diencephalon development” with enrichment strength of 0.73 and 0.67 respectively).

A total of 722 protein–protein interactions were identified between nodes of genes which is more than twice expected at random sample (320 expected) providing protein–protein enrichment p-value < 1.0e-16. 1444 reciprocal interactions were detected (See also Additional file 3: Fig. S3, S4; Additional file 1: Table S7). 380 (40.2%) genes were excluded from gene enrichment and protein–protein interaction analysis due to missing information in the STRING database.

### Previously encountered markers in GH-producing PitNETs

**Fig. 4:**
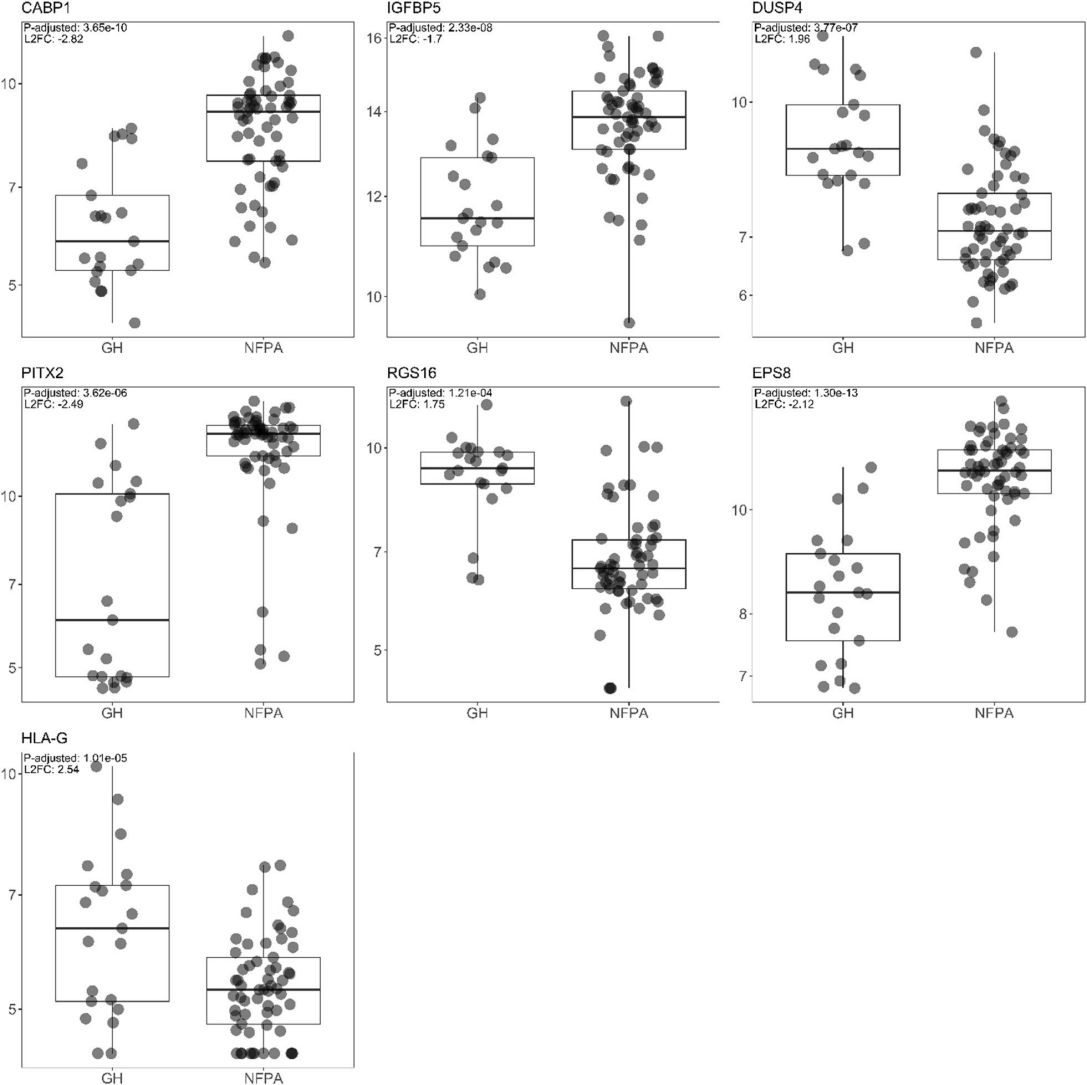
Box plot visualisation of seven proposed candidate genes involved in altered pathway signalling matching with information found in literature for somatotropinoma and non-functioning PitNET subtypes. Gene expression levels transformed with variance stabilising transformation

To test whether our data is an overall representation of the genetic alteration landscape of PitNET tissues, we curated a list of 428 genes associated with genetic changes of said tumour tissue types as found in literature [1]. As a result, 55 genes from the curated list were found to be differentially expressed in our data set, 29 of whom were upregulated with median L2FC of 2.31 (IQR = 1.4) and 26 of whom were downregulated with median L2FC of − 2.43 (IQR = 0.78) (Additional file 1: Table S8.). Genes like calcium binding protein 1 (*CABP1*)*,* insulin like growth factor binding protein 5 (*IGFBP5*)*,* dual specificity phosphatase 4 (*DUSP4*)*,* paired like homeodomain 2 (*PITX2*)*,* and regulator of G protein signalling 16 (*RGS16*) have previously been suggested as candidate markers for PitNETs [1]. Epidermal growth factor receptor pathway substrate 8 (*EPS8*) controls various cellular protrusions by regulating actin cytoskeleton dynamics and architecture as well as participates in growth factor activation, therefore promotes cell proliferation and cell survival within tumour. Overexpression of *EPS8* has previously been detected in PitNETs compared to normal pituitary [42]. Major histocompatibility complex, class I, G (*HLA-G*) has also been detected in various types of PitNETs, primarily in lactotroph PitNETs suggesting tumour immuno-surveillance suggesting tumour immune-surveillance issue [1], (Fig. 4, Additional file 1: Table S8).

### Differential expression in preoperative SSA treatment group

**Fig. 5:**
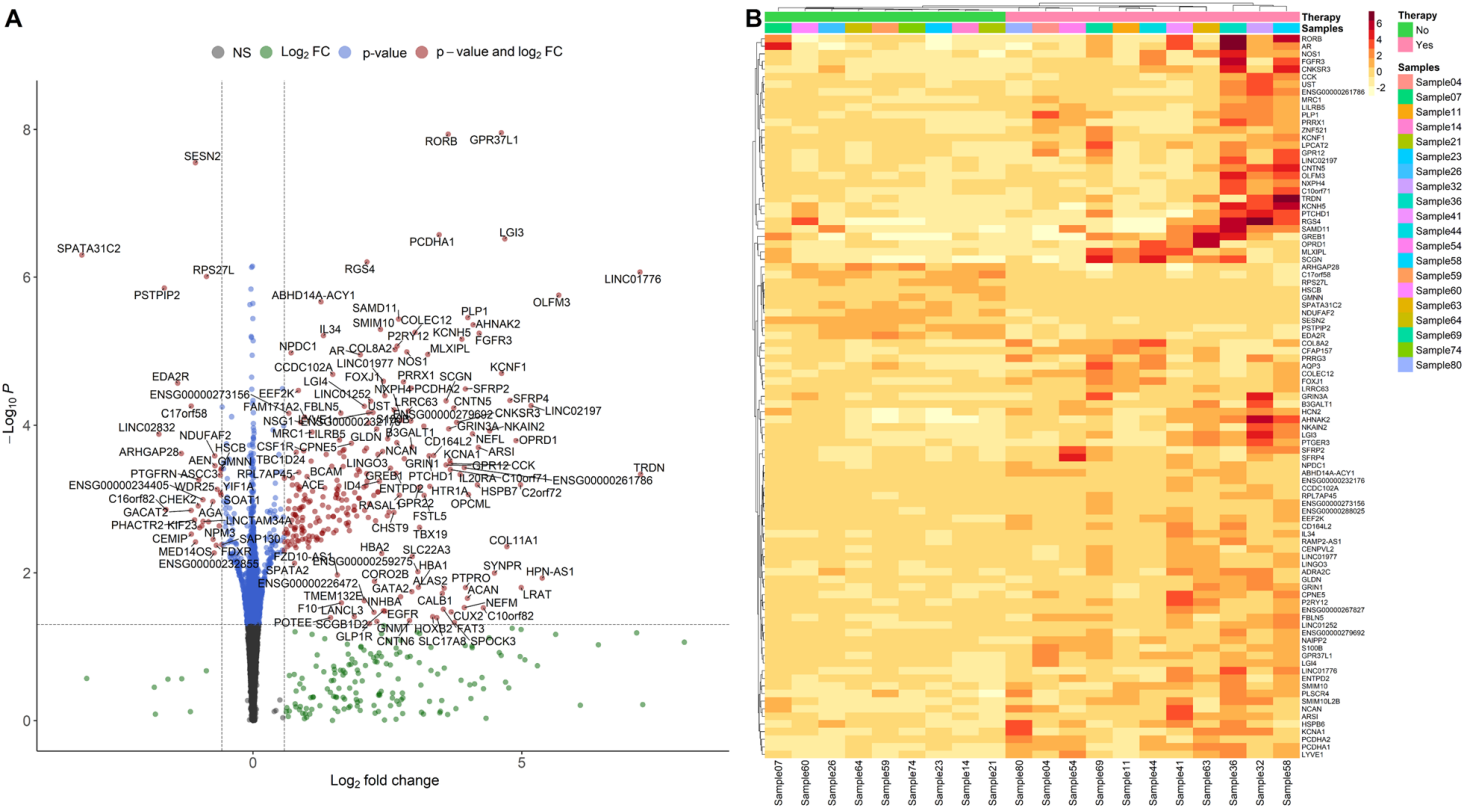
**A**. (left) Volcano plot of differential expression results between PitNET SSA therapy and non-therapy groups with ‘apeglm’ transformed log fold change values. Dashed vertical lines represent absolute log2 fold change threshold of 0.58 and horizontal dashed line represents p-value threshold of 0.05. Red dots mark genes passing both thresholds. **B**. (right) Heatmap visualising gene level and mean normalised expression values of differentially expressed genes across all samples of the treated and untreated groups. Pink bar above the heatmap represents samples treated with SSA therapy, the green bar represents the untreated samples

Frequency filtering (ten counts in at least three samples), left 22348 genes available for further analysis. Parametric dispersion model was determined to have the best fit with this sample set. Differential expression test between GH-producing PitNET with SSA/DA therapy (N = 11) and without therapy (N = 9) initially produced 143 DEG’s with 108 (75.52%) upregulated and 35 (24.48%) downregulated genes in the therapy group. After L2FC shrinkage and results table subsetting with p-adjusted threshold of 0.05 and L2FC threshold of 0.58, a total of 95 DEG’s were obtained. 85 of these DEG’s were upregulated in the therapy group with a median L2FC of 2.68 (IQR = 1.67) and 10 were downregulated with a median L2FC of -1.11 (IQR = 0.63) (Fig. 5, Additional file 1: Table S9).

### Functional enrichment and protein–protein interaction results for SSA treated/untreated GH-producing PitNET comparison

By performing functional enrichment analysis, we obtained three terms associated with biological processes (Gene Ontology), eight with a cellular component (Gene Ontology) and six with annotated keywords (UniProt) with an average enrichment strength of 0.58 (SD = 0.28) DEG’s were revealed to be involvement in regulation of ion transport, generation of neurons and nervous system development with a protein–protein enrichment p-value of 0.0005 (Additional file 1: Table S10, S11, Additional file 3: Fig S5, S6). Enrichment was not possible for 16 (16.8%) DEG’s because of missing information or protein names in the string database.

### Comparison of GH-producing PitNET SSA treated/untreated differential expression results in an unrelated cohort and a curated list of literature

**Fig. 6:**
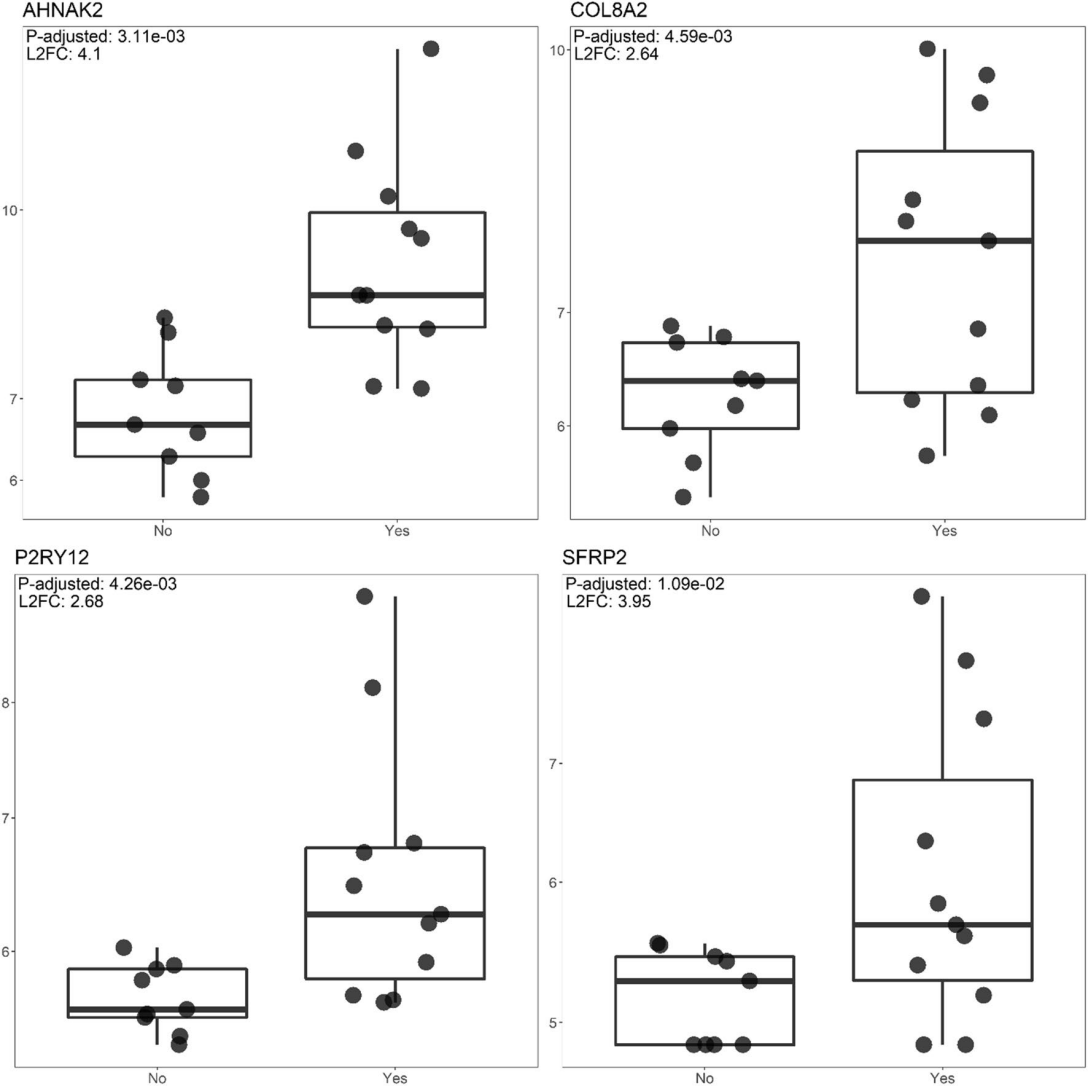
Box plot graphs of four DEG’s from this study overlapping with DEG’s from Saksis et. al. 2021 of the same design and with a curated list of PitNET associated genes. Gene expression values transformed with variance stabilising transformation (Table 1).

Comparison of obtained DEG’s in SSA/DA treatment group to a curated list of PitNET relevant genes and two publications: Saksis et. al and Neou et. al. with the response variable description matching that of this publication: PitNET treatment with SSA therapy or lack of it [1, 11, 17]. Both publications had similar sample sizes for the treatment groups, respectively five for Neou et. al. and six for Saksis et. al, while the non-treated groups of acromegaly patients were 15 and six respectively. In total four DEG’s were found to be overlapping between mentioned sources of data. No overlap was detected between Neou et. al. and the current results. None of the overlapping DEG’s had a differing change of expression: all four were upregulated in the therapy group and downregulated in the untreated group. Notably, our discovery DE results for these four genes had smaller standard error values pointing to more evenly matched contrast group samples (Table 1, Fig. 6).

**Table 1:** DEG’s between SSA treated and untreated GH-producing PitNET groups replicated in an independent sample set and relevant literature

Source	Matching DEG’'s	Gene name	L2FC source	L2FC this study	p-adj. source	p-adj. this study
[17]	AHNAK2	AHNAK nucleoprotein 2	3.38 ± 1.16	4.10 ± 0.61	0.041	0.003
[17]	COL8A2	Collagen type VIII alpha 2 chain	3.26 ± 1.02	2.64 ± 0.74	0.006	0.005
Curated list from literature	P2RY12	Purinergic receptor P2Y12	–	2.68 ± 0.75	–	0.004
Curated list from literature	SFRP2	Secreted frizzled related protein 2	–	3.95 ± 1.23	–	0.011

Finally, to test whether and how effectively the SSA/DA therapy affects the expression of genes upregulated in the GH secreting acromegaly patients, we compared the differential expression results from both groups. Six genes were found to be overlapping (Table 2.). All of the matching differentially expressed genes had an increased expression in the SSA/DA therapy contrast, with the median positive L2FC difference of 3.49 (IQR = 2.79). Most of the observed genes are associated with cell membrane and cell adhesion.

**Table 2:** Six differentially expressed genes overlapping between SSA/DA comparison and GH/NF comparison groups

Gene symbol	Gene name	(GH vs NF)	(GH vs NF)	(Therapy vs non-therapy GH)	(Therapy vs non-therapy GH)
CD164L2	CD164 molecule like 2	− 1.58	0.01	3.36	0.04
CFAP157	Cilia and flagella associated protein 157	1.61	0	1.69	0.04
CPNE5	Copine 5	− 1.95	0	1.83	0.03
FBLN5	Fibulin 5	− 1.56	0	1.64	0.02
NCAN	Neurocan	− 1.97	0	2.68	0.03
PCDHA1	Protocadherin alpha 1	2.35	0	3.46	0

### Transcriptomic patterns of pituisphere model

**Fig. 7:**
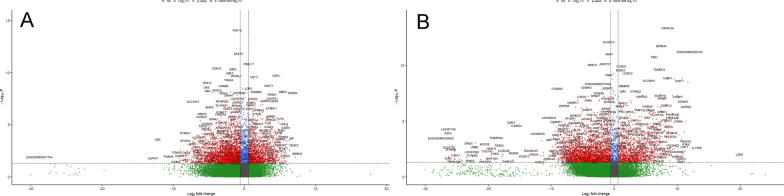
Volcano plot of LRT differential expression results between control and with (**A**) octreotide and (**B**) cabergoline incubated pituispheres. Dashed vertical lines represent absolute log fold change threshold of 0.58 and horizontal dashed line represents p values threshold of 0.05. Red dots represent genes passing both thresholds and grey dots represent dots not passing any of the thresholds

To detect more stable alterations in the pituisphere expression profile we first used time-series differential expression analysis with Likelihood Ratio Test between the control (non-incubated) and incubated with octreotide or cabergoline. groups. Thresholds (L2FC ± 0.58 and p-adjusted < 0.05) revealed 1941 differentially expressed genes for the octreotide and control comparison (Fig. 7A). Majority (1237, (64%) DEG’s were downregulated with a median L2FC of -2.35 (IQR = 2.33) while 703 (36%) were upregulated with a median L2FC of 1.92 (IQR = 1.96) in the incubated pituisphere group (Additional file 1: Table S12). For the cabergoline incubated and non-incubated time-series contrast, a list of 1991 differentially expressed genes was obtained (L2FC ± 0.58 and p-adjusted < 0.05 thresholds). 1186 (59%) DEG’s were downregulated with a median L2FC of − 3.89 (IQR = 3.97) and 805 (41%) were upregulated with a median L2FC of 3.74 (IQR = 4.08) in the incubated PitNET sphere group. (Fig. 7B, Additional file 1: Table S13).

To investigate the more minute perturbations of pituisphere expression profile in response to the incubation length with octreotide or cabergoline, we performed differential expression analysis at each time point (4 h, 8 h, 24 h, 48 h and 72 h) using the Wald test and identified differentially expressed genes after subsetting the data with L2FC ± 0.58 and p-adjusted < 0.05 thresholds. For the octreotide contrasts, most DEGs were found in the PitNET sphere group incubated for 48 h. The majority of DEGs at each time point were downregulated except for the group treated for 72 h (54% of DEGs were upregulated) (Table 3, Additional file 3: Fig. S7–S11.; Additional file 1: Table S15–S19). When testing the incubation length contrasts for cabergoline, we observed that the largest number of DEGs was found in the PitNET sphere group incubated for 72 h. The majority of DEGs at each time point were downregulated except for the group treated for 4 h (65% of DEGs were upregulated) (Additional file 3: Fig. S12–S16; Additional file 1: Table S20–S24) (Table 3).

**Table 3:** Number of differentially expressed genes at each time point treated with octreotide or cabergoline

Incubation time (h)	Octreotide			Cabergoline		
	Count of DEGs	Count of downregulated DEGs (%)	Count of upregulated DEGs (%)	Count of DEGs	Count of downregulated DEGs (%)	Count of upregulated DEGs (%)
4	192	164 (85)	28 (15)	446	157 (35)	289 (65)
8	281	210 (75)	71 (25)	941	538 (57)	403 (43)
24	316	233 (74)	83 (26)	1015	867 (85)	148 (15)
48	493	434 (88)	59 (12)	715	470 (66)	245 (34)
72	163	75 (46)	88 (54)	1293	1033 (80)	260 (20)

**Fig. 8:**
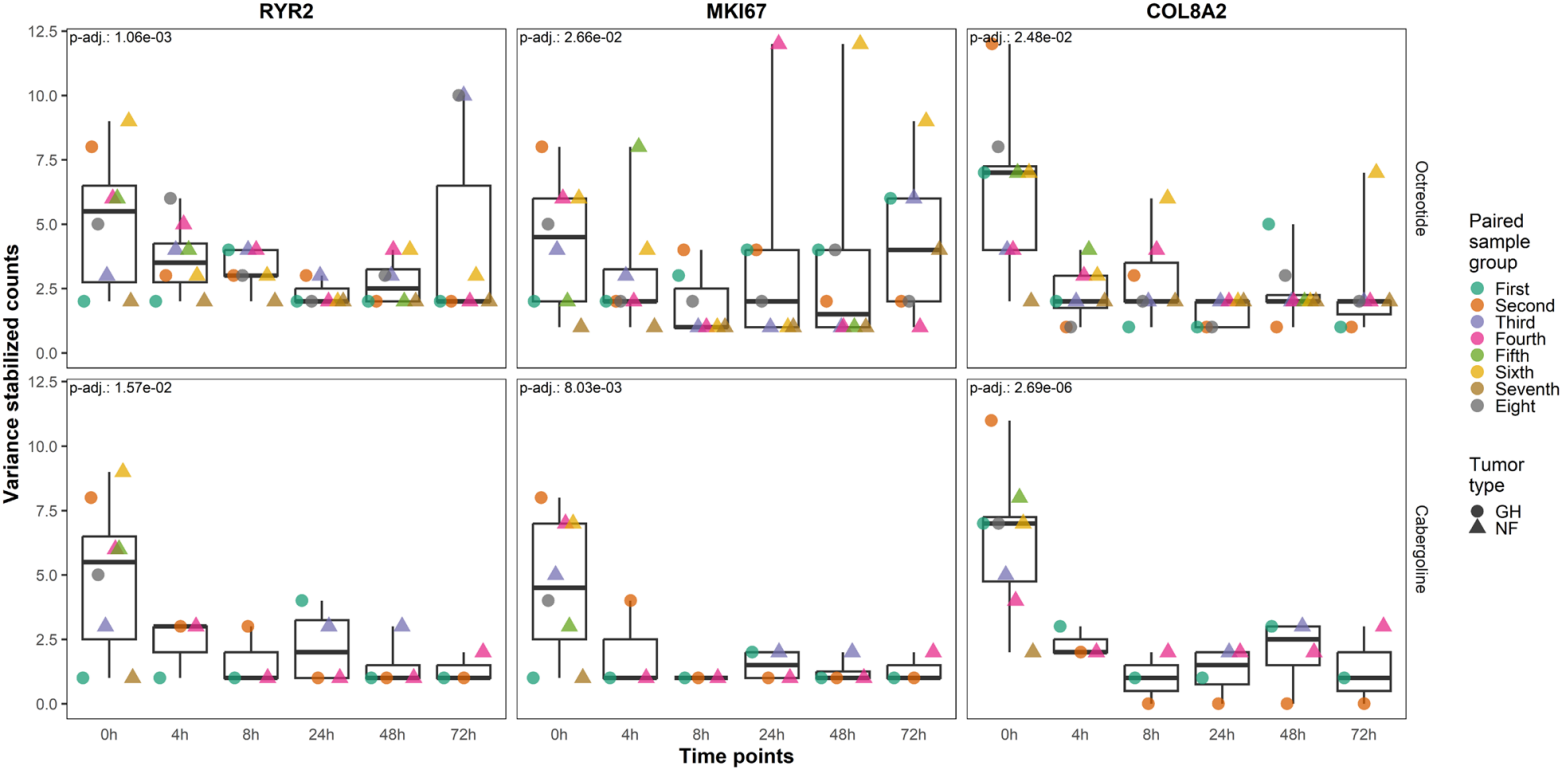
Differentially expressed time stable downregulated genes in the pituispheres incubated with octreotide (**A**), and in the pituispheres incubated with cabergoline **B**. Gene expression values normalized with variance stabilizing transformation

When comparing genes with altered expression from the time-series tests in both octreotide and cabergoline incubated sample groups, we observed that ryanodine receptor 2 (*RYR2*), marker of proliferation KI-67 (*MKI67*), and collagen type VIII alpha 2 chain (*COL8A2*) were downregulated (Fig. 8).

For the three mentioned DEG`s, 59 time-series enrichments were detected in the octreotide incubated group (Additional file 1: Table S25) from the 163 observed in total (Additional file 1: Table S26), and 91 time-series enrichments were detected in the cabergoline incubated group (Additional file 1: Table S27) from the 263 observed in total (Additional file 1: Table S28). We also observed that Major Histocompatibility Complex, Class I, G (*HLA-G*) and Solute Carrier Family 2 Member 1 (*SLC2A1*) genes were downregulated in the presence of octreotide incubating for 4 h and 8 h and for 24 h and 48 h, respectively. Incubating pituispheres with cabergoline for 4 h, 24 h, 48 h, and 72 h (except 8 h) we observed ADP Ribosylation Factor GTPase Activating Protein 1 (*ARFGAP1*) gene downregulation. When incubating pituispheres with cabergoline for 8 h and 24 h, expression alterations of Transforming Growth Factor Beta Receptor 2 (TGFBR2) and Epidermal Growth Factor Receptor Pathway Substrate 8 (EPS8) were noticed, these pathways have been elaborately studied in relation to PitNETs [43–52]. The significance of cadherins in PitNETs was already made clear in the early 2000s [53, 54]. Alterations in the ability of cells to adhere and interact with neighboring cells and extracellular matrix are proven to influence tumour progression and strongly correlate with its aggressiveness [53–55]. All previously mentioned genes were alluded to in the context of PitNETs and are considered as candidate markers for PitNET tumourigenesis.

### Comparison of transcriptome expression profile similarity between matched, untreated samples of PitNET tissue, pituispheres and mesenchymal stromal stem-like cells

**Fig. 9:**
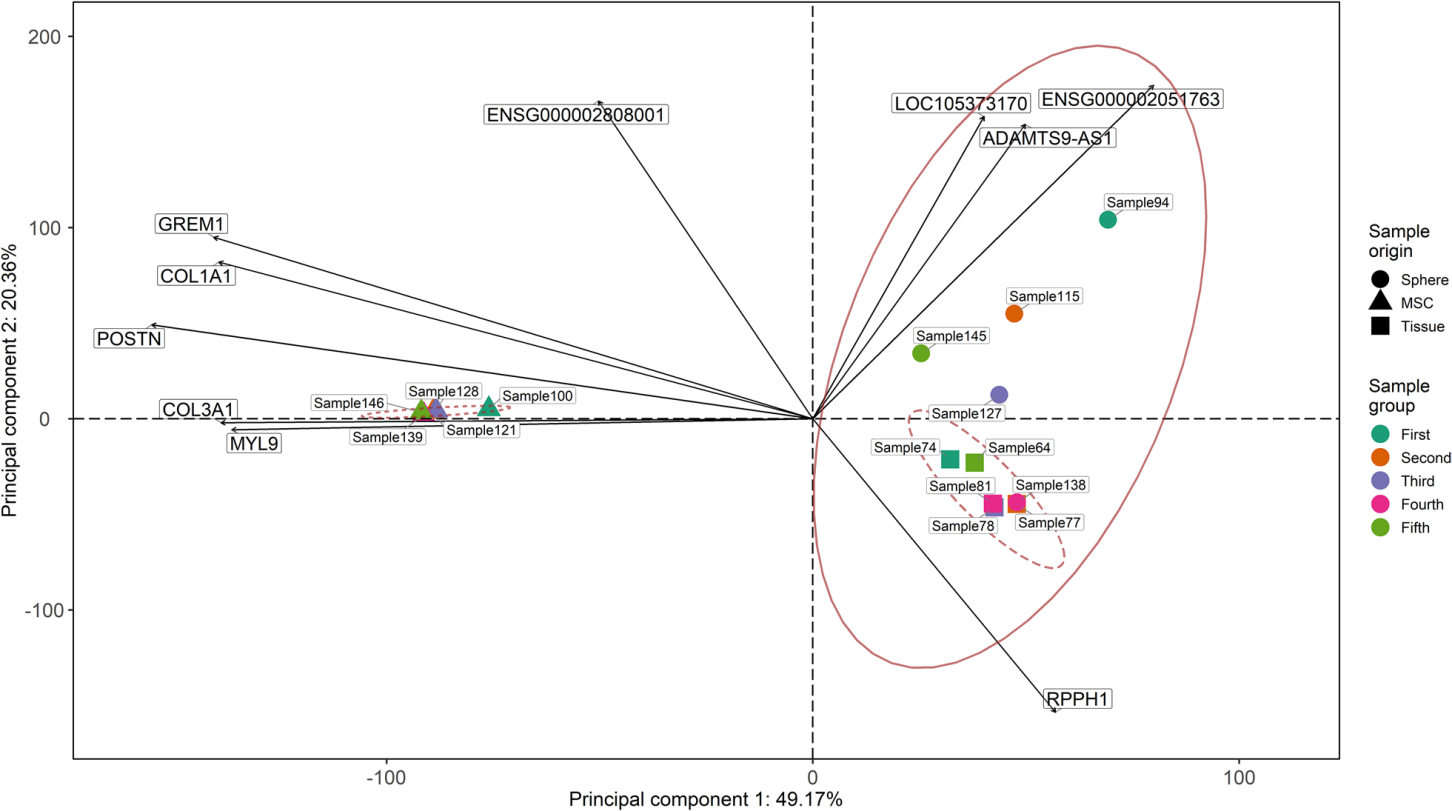
PCA bi-plot for the Euclidean distances of 500 most variable genes amongst PitNET tissue, pituisphere and MSC sample groups. The ellipses represent a 95% confidence level of the multivariate t distribution for each group. Colour indicates biological samples from the same PitNET patient from all three sample origins. Ten gene symbols with the largest loading scores across principal components 1 and 2 are also shown (ENSEMBL notation used for genes with no HGNC symbol). MSC—mesenchymal stromal stem-like cells

To evaluate whether MSC could be used as a potential model for SSA therapy effects on GH-producing PitNETs for future studies, we calculated the Euclidean distances on variance stabilising transformation transformed gene level counts for five PitNET samples involved in the tissue, pituisphere and MSC experiments, resulting in 15 total samples. Euclidean distances were calculated for 500 most variable genes amongst all three sample group origins after filtering genes for frequency to increase gained information. Afterwards the PCA method was applied to said counts to determine the similarity by transcriptional profile distances of mentioned sample groups. Between the compared groups, transcription profile for MSC samples was more dissimilar to PitNET tissue samples, with an explained variance for the first principal component of 49.17%. On the other hand, pituisphere samples overlap PitNET tissue samples almost completely suggesting that they are more similar than MSC samples. Nevertheless, some variation can be observed in the pituisphere group mostly along the second principal component, which explains 20.36% of the variation in the data. Genes with the largest loading scores across principal component 1 are associated with cell extracellular matrix, ion binding, organogenesis and tissue differentiation, while genes with the largest effect on differences between pituisphere and normal tissue are from the lncRNA class (Fig. 9).

### Multiple time point evaluation of octreotide incubated GH3 cells

To investigate whether the effects of octreotide therapy could be observed and replicated in an animal model which also expresses GH1 hormone similar to GH-producing PitNETs, we performed a differential expression test (Wald test) between GH3 cells incubated with octreotide at different time points (4, 8, 24) against unincubated GH3 cells. The test yielded 39 DEG’s (p-adjusted < 0.05) amongst all tested time points, 26 (66.6%) of whom were upregulated and 13 (33.4%) of whom were downregulated (Additional file 3: Figs. S17–S19, Additional file 1: Table S29–S31). At least 60% of DEG’s from each time point comparison were upregulated, gradually increasing along with the incubation time. The number of genes with altered expression also increased along longer incubation periods, indicating that the effects of octreotide incubation not only lasts but, furthermore, increases for at least 24 h (Table 4).

**Table 4:** Summarization of DE results for multiple time point comparisons of octreotide treated vs untreated GH3 cells (p-adjusted < 0.05)

Comparison	Count of DEG’s	Count of DEG’s (upregulated)	Count of DEG’s (downregulated)	Median L2FC upregulated (IQR)	Median L2FC downregulated (IQR)
4 h *vs* control	6	4 (66%)	2 (33%)	0.54 (0.49)	− 0.48 (0.05)
8 h *vs* control	10	8 (80%)	2 (20%)	4.63 (12.35)	− 1.12 (0.63)
24 h *vs* control	23	14 (61%)	9 (39%)	8.95 (13.72)	− 0.27 (0.08)

*DE* differential expression

Next, we checked for Homo sapiens orthologous genes and performed enrichment, and protein–protein interaction analysis for said genes of both organisms to determine pathways and molecular functions potentially affected by detected differentially expressed genes. Only the incubation period of 24 h had a statistically significant number of enrichment terms and protein–protein interactions in Rattus norvegicus, while there were no significant interactions in the human orthologues in any of the time points (Table 5, Additional file 3: Fig. S21–S22; Additional file 1: Table S32–S33).

**Table 5:** Results of functional enrichment and protein–protein interaction for differentially expressed genes in the octreotide incubated GH3 cells in comparison to control in both Rattus norvegicus and Homo sapiens

Comparison	Number of human orthologous genes	Enrichment RN	Reciprocal P-P interactions RN (p-value)	Enrichments HS	Reciprocal P-P interactions HS (p-value)
4 h vs control	3 (50%)	–	–	–	–
8 h vs control	4 (40%)	–	–	–	–
24 h vs control	11 (47%)	2	4 (0.154)	–	2 (0.168)

*RN* Rattus norvegicus, *HS* Homo sapiens

### Comparative pathway analysis

Finally, we performed a pathway analysis by determining which significantly affected pathways were common in each of the characterised groups and subsequently searching for matching DEG’s within the matched pathways as possible time stable drivers for changes associated with PitNET therapy. First, we compared functional enrichment results from the PitNET tissue therapy sample group with the cabergoline incubated PitNET pituispheres. From the 17 enriched pathways of tissue SSA therapy comparison and 939 pathways of the combined cabergoline incubation time point comparison results (Additional file 1: Table S34–S38), nine matching pathways were detected, six of which were categorised as ‘GO Process’ and three as ‘GO Component’. The number of overlapping pathways slightly increased with the incubation time, suggesting that long lasting alterations associated with SSA therapy are established after 24–72 h. Strength for the overlapping pathways was higher in the tissue therapy group (mean 0.52 ± 0.13) compared to the combined cabergoline group (0.2 ± 0.05), indicating that while the long-term effects can be determined after 24–72 h, the number of genes with altered expression increases after application of prolonged therapy (Table 6).

**Table 6:** Overlapping significantly enriched pathways (FDR < 0.05) and genes (L2FC >  ± 0.58, p-adjusted < 0.05) between PitNET tissue therapy and Cabergoline multi-time point incubation pituisphere therapy results

Term	Category	Term description	Strength tissue therapy	FDR tissue therapy	Strength cabergoline	FDR cabergoline	Time	Overlapping genes
GO:0098793	GO Component	Presynapse	0.63	0.049	0.25	0.023	8 h	None
GO:0007399	GO Process	Nervous system development	0.47	0.001	0.16	0.005	24 h	RGS4
GO:0048699	GO Process	Generation of neurons	0.53	0.004	0.21	0.003	24 h	None
GO:0005886	GO Component	Plasma membrane	0.26	0.013	0.11	0.004	48 h	None
GO:0007399	GO Process	Nervous system development	0.47	0.001	0.21	0.0002	48 h	None
GO:0048699	GO Process	Generation of neurons	0.53	0.004	0.28	3.58E-05	48 h	None
GO:0007399	GO Process	Nervous system development	0.47	0.001	0.13	0.023	72 h	EEF2K, RGS4
GO:0043269	GO Process	Regulation of ion transport	0.7	0.004	0.22	0.048	72 h	KCNH5, RGS4
GO:0098793	GO Component	Presynapse	0.63	0.049	0.23	0.026	72 h	None

Within the overlapping pathways of tissue therapy and cabergoline incubation contrasts, three distinct genes were found to be driving described changes—*RGS4* (Regulator of G protein signalling 4), *EEF2K* (Eukaryotic elongation factor 2 kinase) and *KCNH5* (Potassium voltage-gated channel subfamily H member 5). *RGS4* is consistent in its expression upregulation across tissue and pituisphere samples at different octreotide incubation periods, unleash *EEF2K* and *KCNH5*, which are upregulated in SSA incubated tissue samples, but downregulated in the octreotide incubated pituispheres.

On the other hand, when comparing PitNET tissue SSA therapy functional enrichment results (strength 0.63, FDR 0.05) with pituispheres combined octreotide incubation time point (strength 0.41, FDR 0.04) enrichment results (Additional file 1: Table S39–S42), only one pathway was found to be in both groups—‘Presynapse’, which specifically was detected at the octreotide 24 h incubation time point. Again, similarly to comparison of tissue SSA therapy and pituisphere cabergoline enrichment results, a larger number of DEGs was driving the enrichment in the tissue therapy group, supporting the assumption that a longer period of SSA therapy results in a larger number of genes with altered expression.

Pathway comparison of PitNET tissue SSA therapy enrichment results was also performed for the multiple time point results of octreotide incubated GH3 cells. Due to the low number of differentially expressed genes in the GH3 sample group, with even fewer identified as human orthologues none of them overlapped with PitNET tissue SSA therapy enriched pathways.

## Discussion

In this study we investigated the transcriptomic landscape of GH-producing PitNETs to identify characteristic markers and follow alterations of the expression profiles induced by clinically used drugs in tumour tissue and available cell models. We have shown that GH-producing PitNETs have transcriptomic profiles with distinct perturbed growth hormone related pathways consistent to its functional status alongside changes in inner cell signalling, ion transport, cell adhesion and extracellular matrix characteristic pathways. Furthermore, we have provided additional evidence that pituispheres of primary cultures have higher similarity to the actual tumour tissue compared to MSCs which would limit MSC usability as a precise model for PitNET studies. Using the pituispheres we elucidated that treatment regimens (octreotide and cabergoline) affect specific cell proliferation gene expression (*MKI67)* and expression of members of core functionality pathways (*RYR2*, *COL8A2*, H*LA-G*, *SLC2A1*, *ARFGAP1*, *TGFBR2)*. While using commercial GH3 cell line we observed that medication did not have transcriptomic effects similar to preoperative treatment in PitNET tissue or pituisphere model.

During the GH-producing PitNET transcriptome analysis we were able to identify 1595 DEGs that distinguished this group from non-functional PitNET, the expected result was that growth hormone related pathways were upregulated in GH-producing group, but alongside that we also detected other candidates among these only 41 have been mentioned in transcriptomic studies of PitNETs, therefore, there are distinct patterns that characterise GH-producing PitNETs that could be used for better understanding of tumour biology and development of management strategies [1]. We observed a large share of candidate gene expression implicated in ion flux regulation and inner cell signalling which is in concordance that these cells are metabolically active and producing large amounts of molecules for secretion.

Noteworthy, our pituisphere experiments showed that incubation in octreotide and cabergoline reduce expression levels of Ki-67 (*MKI67*) which is one of widely used markers in histology to assess tumour proliferation capacity therefore also aggressiveness [56]. This indicates that the pituisphere model is also affected by treatment in accordance with tumour biology *in-vivo*. Ki-67 has been previously downregulated in pre-operatively treated patients’ PitNETs [11]. On the other hand, we did not observe any significant downregulation of Ki-67 in medication treated GH3 cell line, for which further confirmation is needed but it indicates that pituispheres have similar response to drugs as PitNETs themselves.

In both modalities of pituisphere model (incubated in octreotide or cabergoline) we observed downregulation of *RYR2,* also slight upregulation of *RYR1* and *RYR3* was observed in GH-producing PitNET analysis (Additional file 1: Table S2). Altered expression of this factor has been found before in several transcriptomic studies of PitNET gene expression [51, 57, 58]. While another member of the ryanodine receptor family: *RYR1* has been reported as carrier of somatic variants in at least three PitNET studies [21, 59, 60]. Ryanodine receptors (*RYR*s) are Ca^2+^ intracellular channels located in the endoplasmic reticulum (ER) being one of main triggers for signalling transduction coupled to calcium release from ER. These receptors have been implicated in the development of various human diseases with vascular or neuronal components—heart failure, arrhythmia, myopathies and neurodegenerative disorders [61, 62]. Calcium signalling has been previously linked to crosstalk with other signalling pathways of PitNET tumours therefore further studies of *RYR* family involvement in pathogenesis needs to be carried out [63, 64]. *RYR*s have been implicated in gonadotropin-releasing hormone signalling where in rat models the mRNA levels of *RYR*s have been found to regulated by gonadotropins, this opens novel interesting investigation window also for PitNET functional studies [65].

Several overlapping candidates related to neuronal system and ion channel regulation were observed in a group of cabergoline incubated pituispheres and pre-operatively treated patients’ PitNET (Table 6). As expected, we observed expressional changes of factor regulator of G protein signalling 4 (RGS4) that act by activating GTPase activating proteins (GAPs) that are crucial triggers in transmitting signals via G protein coupled receptors (GPCRs). This is not surprising as octreotide is targeting SSTRs and cabergoline D2R both belonging to GPCRs, treatment have been previously already reported to downregulate SSTR expression and our study helps to highlight specific factors involved in intracellular adaptation loop [66, 67]. Previously, only RGS16, also an RGS family member, has been mentioned in transcriptomic studies of PitNETs [68, 69]. Downstream signalling of SSTRs upon SSA stimulation have been widely studied in relation to potential SSA drug resistance mechanisms and primary roles of beta-arrestins and cytoskeleton protein filamin A have been investigated, however, the role of RGS candidates involvement in drug induced feed-back mechanism could also be investigated [70, 71]. We noticed slight upregulation of RGS7, 8, 16 and 17 in GH-producing PitNET transcriptomic results (Additional file 1: Table S2), but how this could be related to GH-producing functionality needs to be investigated.

Other overlapping factors found both in pituispheres and tissue analysis (Table 6) were eukaryotic elongation factor 2 kinase (EEF2K) and potassium voltage-gated channel subfamily H member 5 (*KCNH5*). According to Human Protein Atlas EEF2K is a protein kinase involved in downregulation of translation elongation, many studies have indicated the role of EEF2K in tumourigenesis and the role of EEF2K as anticancer drug target have been discussed, but so far, we have not found previous evidence of its involvement in PitNETs [72].

*KCNH5* belongs to the family of ion channels that are involved in neurotransmitter and hormone release, that could be functionally linked to cell signal transduction pathways related to PitNET medication use. *KCNH5* is predominantly expressed in various parts of the brain, but in smaller levels it has been detected also in adrenal and pituitary glands [73]. It has been demonstrated that in *Cxcr2* knockout mice pituitary functions are altered and *KCNH5* related pathways are downregulated, but how this could be related to PitNET development or response to therapy needs to be investigated [74].

Our data strongly supports evidence that extracellular matrix (ECM) is involved in molecular patterning of PitNET functionality and response to medical treatment. There is previous evidence that PitNETs can contain fibrotic tissue composed of collagens and alteration on collagen composition can have antitumoural effects [75–77]. It has also been demonstrated that tumours from patients pre-treated with SSA have softer tumour consistency it is not clear whether this could be related to the changes in ECM caused by medication [78, 79]. We have already previously observed collagens and other ECM factor modulation in PitNETs treated with SSA/DA [17]. In this study, we found significant downregulation of *COL8A2* in octreotide incubated pituispheres, but in previous reports and our extended results here, *COL8A2* was upregulated in patients with preoperative therapy. This indicates some consistency in tissue data but discrepancy with pituispheres, that could be explained by regulation of ECM also by inner biological environment of the tumour that is different from pituisphere propagation media (Table 7).

**Table 7:** Matching differentially expressed genes in detected significant enrichment terms between the PitNET tissue SSA therapy and combined cabergoline incubated PitNET pituisphere sample groups (DEG’s: L2FC >  ± 0.58, p-adjusted < 0.05; Enrichment terms: FDR < 0.05)

Incubation period	Enriched term	Gene	LF2C tissue ± SE	L2FC pituisphere ± SE
24 h	Nervous system development	RGS4	2.12 ± 1.87	1.14 ± 1.99
72 h	Nervous system development	EEF2K	0.84 ± 0.26	− 4.90 ± 2.71
72 h	Nervous system development	RGS4	2.12 ± 1.87	1.26 ± 2.15
72 h	Regulation of ion transport	KCNH5	3.89 ± 1.10	− 0.63 ± 1.34
72 h	Regulation of ion transport	RGS4	2.12 ± 1.87	1.26 ± 2.15

While we observed some overlap between our discovery cohort, [17] and the literature, the lack of co-occurring differentially expressed genes with the validation cohort from Neou et. al. is not surprising, given the fact that the compared subsample groups of SSA/DA therapy and non-therapy are of different sizes, resulting in differing statistical power, while such factors as sequencing technology, laboratory and sample level related batch effect could also play a major role [80].

We also wanted to compare drug effects on transcriptomic patterns of widely used rat cell model GH3 to pituispheres and tumour tissue of pre-operatively treated patients. To our surprise we did not observe similar patterns suggesting that at least on transcriptomic level these cell models differ considerably, and every experimental procedure needs to take into account this possibility when translating and extrapolating results of each model system. For our study we studied GH3 cell line that is widely used in PitNET research and was available in our cell culture facility [19, 20]. It would be interesting to specifically assess also other cell line models by transcriptomic analysis and compare the obtained results. For example, it would be specifically valuable to evaluate transcriptomic landscape of GH4C1 that compared to GH3 cells produce lower level of GH but higher of PRL and initially have been derived from the same GH3 clone [19]. This could help to assess transcriptomic changes in context of several cell line models and could help researchers in future to select more appropriate investigation model. We also propose that in future single cell transcriptome sequencing of spatial transcriptomics could be used to dissect complexity of various PitNET cell types and deduct their impact on functional experiments. Our study along with others highlights the added value of using NGS for tracing functionality of acquired primary cultures and these novel techniques could bring a novel dimension to PitNET model studies.

We consider that the most significant limitation of this study was the use of non-functional PitNETs as contrast for deduction of GH-producing PitNET transcriptome patterns. According to WHO Classification published in 2017 detailed histological characterization of PitNETs in subgroups according to cell lineage development transcription factors (SF-1, Tpit, Pit-1) is advised [81]. As our sample set dates back to 2010 our sample group included samples without proper assessment of these characteristics, nonetheless we specifically investigated patterns of GH-producing PitNETs compared to hormonally non-functional and non-secreting tumour groups and concentrated on therapy effects altering gene expression. We are well aware that our non-functional PitNET group is most likely heterogeneous that could affect the presented results, however, other molecular factors for example presence of genetic variants in USP8 and GNAS could introduce distinct expression patterns in affected tumours and wider scope on influential factors could affect groups selection alongside WHO PitNET Classification system [11].

## Conclusions

We demonstrate that GH-producing PitNETs have distinct transcriptomics not only on growth hormone signalling pathways, but also in GH-producing PitNET functionality supportive signalling and regulatory pathways: ion transport, extracellular remodelling and calcium signalling. Our data indicate that pituispheres more closely represent transcriptomic profiles of tumour tissue while MSC have significantly altered gene expression. We show that pituispheres treated with octreotide and cabergoline are not directly comparable with pre-operatively treated patients’ tumour tissue, but both share similar patterns of transcriptomic alterations. Therapeutic effects on model cell line GH3 did not match the gene expression changes observed in tumour tissue nor pituispheres. This study highlights the importance of cell transcriptomic profiling for correct model system selection and data interpretation that could be achieved in future by incorporating NGS methods and detailed cell omics profiling in PitNET model research.

## Supplementary Information

**Additional file 1: Table S1**. Patient clinical data and medical treatment information. Differentially expressed genes between non-functioning PitNET and growth hormone secreting PitNET types. **Table S2**. Enrichment results for non-functioning PitNET and growth hormone secreting PitNET type differentially expressed genes obtained from STRING-db (v11.5) (Sorted by decreasing enrichment strength, FDR < 0.05). **Table S3**. Summary of enrichment results for non-functioning PitNET and growth hormone secreting PitNET type from STRING-db(v11.5) (Sorted alphabetically by compartment, FDR < 0.05). **Table S4**. Growth hormone associated enrichment results for non-functioning PitNET and growth hormone secreting PitNET type from STRING-db(v11.5). **Table S5**. Molecule transport channel associated enrichment results for non-functioning PitNET and growth hormone secreting PitNET type from STRING-db(v11.5) (Sorted by enrichment strength, FDR < 0.05). **Table S6**. Protein-protein interaction matrix from non-functioning PitNET and growth hormone secreting PitNET type with interaction scores represented for each interaction type. **Table S7**. Detected differentially expressed genes from non-functioning PitNET and growth hormone secreting PitNET type comparison overlaping with a curated literature list of 236. **Table S8**. Differentially expressed genes between SSA treated/untreated somatotrophin secreting PitNET tissue groups. **Table S9**. Functional enrichment results for differentially expressed genes between SSA treated/untreated PitNET somatotrophin groups. **Table S10**. Protein-Protein interaction results for differentially expressed genes between SSA treated/untreated PitNET GH groups (FDR < 0.05). **Table S11**. Differentially expressed genes between octreotide treated pituispheres at all time points (time-series, LRT) and untreated control (LRT, Sorted by P-adjusted values, L2FC > +/- 0.58, P-adjusted < 0.05). **Table S12**. Differentially expressed genes between cabergoline treated pituispheres at all time points (time-series, LRT) and untreated control. **Table S13**. Differentially expressed genes between cabergoline treated pituispheres after incubation of 8h and untreated control (Wald test, Sorted by P-adjusted values, L2FC > +/- 0.58, P-adjusted < 0.05). **Table S14**. Differentially expressed genes between cabergoline treated pituispheres at all time points (time-series, LRT) and untreated control (LRT, Sorted by P-adjusted values, L2FC > +/- 0.58, P-adjusted < 0.05). **Table S15**. Differentially expressed genes between octreotide treated pituispheres after incubation of 4h and untreated control. **Table S16**. Differentially expressed genes between octreotide treated pituispheres after incubation of 8h and untreated control (Wald test, Sorted by P-adjusted values, L2FC > +/- 0.58, P-adjusted < 0.05). **Table S17**. Differentially expressed genes between octreotide treated pituispheres after incubation of 24h and untreated control. **Table S18**. Differentially expressed genes between octreotide treated pituispheres after incubation of 48h and untreated control (Wald test, Sorted by P-adjusted values, L2FC > +/- 0.58, P-adjusted < 0.05). **Table S19**. Differentially expressed genes between octreotide treated pituispheres after incubation of 72h and untreated control (Wald test, Sorted by P-adjusted values, L2FC > +/- 0.58, P-adjusted < 0.05). **Table S20**. Differentially expressed genes between cabergoline treated pituispheres after incubation of 4h and untreated control (Wald test, Sorted by P-adjusted values, L2FC > +/- 0.58, P-adjusted < 0.05). **Table S21**. Differentially expressed genes between cabergoline treated pituispheres after incubation of 8h and untreated control. **Table S22**. Differentially expressed genes between cabergoline treated pituispheres after incubation of 24h and untreated control. **Table S23**. Differentially expressed genes between cabergoline treated pituispheres after incubation of 48h and untreated control (Wald test, Sorted by P-adjusted values, L2FC > +/- 0.58, P-adjusted < 0.05). **Table S24**. Differentially expressed genes between cabergoline treated pituispheres after incubation of 72h and untreated control (Wald test, Sorted by P-adjusted values, L2FC > +/- 0.58, P-adjusted < 0.05). **Table S25**. Functional enrichment results for differentially expressed RYR2, COL8A2 and MKI67 genes in the octreotide time-series comparison (Results sorted by decreasing enrichment strength, FDR < 0.05). **Table S26**. Functional enrichment results for differentially expressed genes in the octreotide time-series comparison (Results sorted by decreasing enrichment strength, FDR < 0.05). **Table S27**. Functional enrichment results for differentially expressed RYR2, COL8A2 and MKI67 genes in the cabergoline time-series comparison (Results sorted by decreasing enrichment strength, FDR < 0.05). **Table S28**. Functional enrichment results for differentially expressed genes in the cabergoline time-series comparison (Results sorted by decreasing enrichment strength, FDR < 0.05). **Table S29**. Differentially expressed genes between octreotide treated GH3 cells after incubation of 4h and untreated control (Wald test, Sorted by P-adjusted values, P-adjusted < 0.05). **Table S30**. Differentially expressed genes between octreotide treated GH3 cells after incubation of 8h and untreated control (Wald test, Sorted by P-adjusted values, P-adjusted < 0.05). **Table S31**. Differentially expressed genes between octreotide treated GH3 cells after incubation of 24h and untreated control (Wald test, Sorted by P-adjusted values, P-adjusted < 0.05). **Table S32**. Functional enrichment results for differentially expressed genes in the GH3 24h vs GH3 control comparison (Results sorted by decreasing enrichment strength, FDR < 0.05). **Table S33**. Protein-Protein interaction results for differentially expressed human orthologue genes between GH3 octreotide treated/untreated 24h incubation period groups (FDR < 0.05). **Table S34**. Functional enrichment results for cabergoline treated pituispheres after incubation of 4h and untreated control (FDR < 0.05). **Table S35**. Functional enrichment results for cabergoline treated pituispheres after incubation of 8h and untreated control (FDR < 0.05). **Table S36**. Functional enrichment results for cabergoline treated pituispheres after incubation of 24h and untreated control (FDR < 0.05). **Table S37**. Functional enrichment results for cabergoline treated pituispheres after incubation of 48h and untreated control. **Table S38**. Functional enrichment results for cabergoline treated pituispheres after incubation of 72h and untreated control (FDR < 0.05). **Table S39**. Functional enrichment results for octreotide treated pituispheres after incubation of 8h and untreated control. **Table S40**. Functional enrichment results for octreotide treated pituispheres after incubation of 24h and untreated control. **Table S41**. Functional enrichment results for octreotide treated pituispheres after incubation of 48h and untreated control. **Table S42**. Functional enrichment results for octreotide treated pituispheres after incubation of 72h and untreated control (FDR < 0.05).

**Additional file 2: TableS1**. Spheroid sample information in regards to origin tissue type and incubation period. **TableS2**. Grouped MSC sample information in regards to sample origin, origin tissue type and sample pairs. **TableS3**. Rattus norvegicus GH3 cell octreotide incubation information in relation to incubation medicine and period.

**Additional file 3: Figure S1**. Variance stabilization transformed heatmap of mean normalized gene level counts for DEGs of the sompatotroph and non-secreting PitNET type comparison. Pink fields in the “Subtype” column row, represent the non-secreting group samples, green fields represent the non-secreting group samples. **Figure S2**. Summarization of the categories in enrichment analysis results for the PitNET non-secreting and somatotroph type comparison. Each bar represents an enrichment category with the corresponding occurance in enrichment results. **Figure S3**. A STRING-db network for the DEG proteins of somatotroph and non-secreting PitNET type comparison. Line thickness indicates confidence of protein interaction. Disconnected nodes were hidden. Text mining disabled as interaction type. **Figure S4**. ASTRING -db network for the DEG proteins of somatotroph and non-secreting PitNET type comparison. Line colour indicates protein interaction type and line count indicates number of interactions. Disconnected nodes were hidden. Text mining disabled as interaction type. **Figure S5**. ASTRING -db network for the DEG proteins of SSA treated/untreted somatotroph PitNET comparison. Line thickness indicates interaction confidence. Disconnected nodes were hidden. Text mining disabled as interaction type. **Figure S6**. A STRING-db network for the DEG proteins of SSA treated/untreted somatotroph PitNET comparison. Line colour indicated interaction type and line count indicates number of interactions. Disconnected nodes were hidden. Text mining disabled as interaction type. **Figure S7**. Variance stabilization transformed heatmap of mean normalized gene level counts for DEGs of the octreotide incubated pituispheres cell and pituisphere control comparsion at 4 hours. Bright green (middle) represents samples from the octreotide 4h incubation period, darker green represents the unincubated pituisphere control samples. **Figure S8**. Variance stabilization transformed heatmap of mean normalized gene level counts for DEGs of the octreotide incubated pituispheres cell and pituisphere control comparsion at 8 hours. Dark green represents the unincubated pituisphere control samples, bright green represents the octreotide incubated pituispheres at 8 hours. **Figure S9**. Variance stabilization transformed heatmap of mean normalized gene level counts for DEGs of the octreotide incubated pituispheres cell and pituisphere control comparsion at 24 hours. Dark green represents the unincubated pituisphere control samples, bright green represents the octreotide incubated pituispheres at 24 hours. **Figure S10**. Variance stabilization transformed heatmap of mean normalized gene level counts for DEGs of the octreotide incubated pituispheres cell and pituisphere control comparsion at 48 hours. Dark green represents the unincubated pituisphere control samples, bright green represents the octreotide incubated pituispheres at 48 hours. **Figure S11**. Variance stabilization transformed heatmap of mean normalized gene level counts for DEGs of the octreotide incubated pituispheres cell and pituisphere control comparsion at 72 hours. Dark green represents the unincubated pituisphere control samples, bright green represents the octreotide incubated pituispheres at 72 hours. **Figure S12**. Variance stabilization transformed heatmap of mean normalized gene level counts for DEGs of the cabergoline incubated pituispheres cell and pituisphere control comparsion at 4 hours. Pink represents the unincubated pituisphere control samples, orange represents the octreotide incubated pituispheres at 4 hours. **Figure S13**. Variance stabilization transformed heatmap of mean normalized gene level counts for DEGs of the cabergoline incubated pituispheres cell and pituisphere control comparsion at 8 hours. Pink represents the unincubated pituisphere control samples, orange represents the octreotide incubated pituispheres at 8 hours. **Figure S14**. Variance stabilization transformed heatmap of mean normalized gene level counts for DEGs of the cabergoline incubated pituispheres cell and pituisphere control comparsion at 24 hours. Pink represents the unincubated pituisphere control samples, orange represents the octreotide incubated pituispheres at 24 hours. **Figure S15**. Variance stabilization transformed heatmap of mean normalized gene level counts for DEGs of the cabergoline incubated pituispheres cell and pituisphere control comparsion at 48 hours. Pink represents the unincubated pituisphere control samples, orange represents the octreotide incubated pituispheres at 48 hours. **Figure S16**. Variance stabilization transformed heatmap of mean normalized gene level counts for DEGs of the cabergoline incubated pituispheres cell and pituisphere control comparsion at 72 hours. Pink represents the unincubated pituisphere control samples, orange represents the octreotide incubated pituispheres at 72 hours. **Figure S17**. Variance stabilization transformed heatmap of mean normalized gene level counts for DEGs of the octreotide incubated GH3 cell and GH3 control comparsion at 4 hours. Light blue fields in the “Time” column row, represent the unincubated control samples, orange fields represent the GH3 samples incubated with octreotide at 4 hours. **Figure S18**. Variance stabilization transformed heatmap of mean normalized gene level counts for DEGs of the octreotide incubated GH3 cell and GH3 control comparsion at 8 hours. Light blue fields in the “Time” column row, represent the unincubated control samples, orange fields represent the GH3 samples incubated with octreotide at 8 hours. **Figure S19**. Variance stabilization transformed heatmap of mean normalized gene level counts for DEGs of the octreotide incubated GH3 cell and GH3 control comparsion at 24 hours. Light blue fields in the “Time” column row, represent the unincubated control samples, orange fields represent the GH3 samples incubated with octreotide at 24 hours. **Figure S20**. A STRING-db network for the DEG proteins of octreotide incubated GH3 cell and GH3 control comparsion for the incubation period of 24 hours. Line thickness indicates interaction confidence. Disconnected nodes were hidden. Text mining disabled as interaction type. **Figure S21**. A STRING-db network for the DEG proteins of octreotide incubated GH3 cell and GH3 control comparsion for the incubation period of 24 hours. Line colour indicated interaction type and line count indicates number of interactions. Disconnected nodes were hidden. Text mining disabled as interaction type.

## Data Availability

The datasets generated and/or analysed during the current study are available in the Gene Expression Omnibus repository, (https://www.ncbi.nlm.nih.gov/geo/query/acc.cgi?acc=GSE200175).

## Abbreviations

PitNET: Pituitary neuroendocrine tumors
DEG: Differentially expressed genes
L2FC: Log2 fold change
SSA: Somatostatin analogue
DA: Dopamine analogue
SE: Standard error
p-adj: Adjusted p value
MSC: Mesenchymal stem cells
WHO: World Health Organization
GH: Growth hormone
PRL: Prolactin
ACTH: Adrenocorticotropic hormone
TSH: Thyroid-stimulating hormone
LH: Luteinizing
FSH: Follicle-stimulating
SSTR: Somatostatin binding receptors
bFGF: Basic fibroblast growth factor
FBS: Fetal bovine serum
ATCC: American type culture collection
rRNA: Ribosomal RNA
LRT: Likelihood ratio test
IQR: Interquartile range
FDR: False discovery rate
GO: Gene ontology
SD: Standard deviation
NF: Non functioning
PCA: Principal component analysis
lncRNA: Long non-coding RNA
HGNC: HUGO gene nomenclature committee
DE: Differential expression

## References

[1] Peculis R, Niedra H, Rovite V (2021). Large scale molecular studies of pituitary neuroendocrine tumors: novel markers mechanisms and translational perspectives. Cancers.

[2] Feola T, Carbonara F, Verrico M, Di Crescenzo RM, Gianno F, Colonnese C (2022). Immunotherapy for aggressive and metastatic pituitary neuroendocrine tumors (PitNETs): State-of-the Art. Cancers.

[3] Asa SL, Mete O, Perry A, Osamura RY (2022). Overview of the 2022 WHO classification of pituitary tumors. Endocr Pathol.

[4] Trouillas J, Jaffrain-Rea M-L, Vasiljevic A, Raverot G, Roncaroli F, Villa C (2020). How to classify pituitary neuroendocrine tumors (PitNET)s in 2020. Cancers.

[5] Chin SO (2020). Epidemiology of functioning pituitary adenomas. Endocrinol Metab.

[6] Yavropoulou MP, Tsoli M, Barkas K, Kaltsas G, Grossman A (2020). The natural history and treatment of non-functioning pituitary adenomas (non-functioning PitNETs). Endocr Relat Cancer.

[7] Molitch ME (2017). Diagnosis and treatment of pituitary adenomas. JAMA.

[8] Cakir M, Dworakowska D, Grossman A (2010). Somatostatin receptor biology in neuroendocrine and pituitary tumours: Part 1—molecular pathways. J Cell Mol Med.

[9] Peverelli E, Treppiedi D, Giardino E, Vitali E, Lania AG, Mantovani G (2015). Dopamine and somatostatin analogues resistance of pituitary tumors: focus on cytoskeleton involvement. Front Endocrinol.

[10] Franck SE, Muhammad A, van Lely AJ, Neggers SJCMM (2016). Combined treatment of somatostatin analogues with pegvisomant in acromegaly. Endocrine.

[11] Neou M, Villa C, Armignacco R, Jouinot A, Raffin-Sanson M-L, Septier A (2020). Pangenomic classification of pituitary neuroendocrine tumors. Cancer Cell.

[12] Taniguchi-Ponciano K, Andonegui-Elguera S, Peña-Martínez E, Silva-Román G, Vela-Patiño S, Gomez-Apo E (2020). Transcriptome and methylome analysis reveals three cellular origins of pituitary tumors. Sci Rep.

[13] Kim YH, Kim JH (2019). Transcriptome analysis identifies an attenuated local immune response in invasive nonfunctioning pituitary adenomas. Endocrinol Metab.

[14] Li J, Qian Y, Zhang C, Wang W, Qiao Y, Song H (2021). LncRNA LINC00473 is involved in the progression of invasive pituitary adenoma by upregulating KMT5A via ceRNA-mediated miR-502-3p evasion. Cell Death Dis.

[15] Wang W, Xu Z, Fu L, Liu W, Li X (2014). Pathogenesis analysis of pituitary adenoma based on gene expression profiling. Oncol Lett.

[16] Beylerli O, Khasanov D, Gareev I, Valitov E, Sokhatskii A, Wang C (2021). Differential non-coding RNAs expression profiles of invasive and non-invasive pituitary adenomas. Noncoding RNA Res.

[17] Saksis R, Silamikelis I, Laksa P, Megnis K, Peculis R, Mandrika I (2021). Medication for acromegaly reduces expression of MUC16, MACC1 and GRHL2 in pituitary neuroendocrine tumour tissue. Front Oncol.

[18] Guo J, Fang Q, Liu Y, Xie W, Zhang Y, Li C (2021). Identifying critical protein-coding genes and long non-coding RNAs in non-functioning pituitary adenoma recurrence. Oncol Lett.

[19] Ooi GT, Tawadros N, Escalona RM (2004). Pituitary cell lines and their endocrine applications. Mol Cell Endocrinol.

[20] Zhu Z, Cui W, Zhu D, Gao N, Zhu Y (2020). Common tools for pituitary adenomas research: cell lines and primary cells. Pituitary.

[21] Peculis R, Mandrika I, Petrovska R, Dortane R, Megnis K, Nazarovs J (2020). Pituispheres contain genetic variants characteristic to pituitary adenoma tumor tissue. Front Endocrinol.

[22] Xu Q, Yuan X, Tunici P, Liu G, Fan X, Xu M (2009). Isolation of tumour stem-like cells from benign tumours. Br J Cancer.

[23] Mertens F, Gremeaux L, Chen J, Fu Q, Willems C, Roose H (2015). Pituitary tumors contain a side population with tumor stem cell-associated characteristics. Endocr Relat Cancer.

[24] Vankelecom H, Roose H (2017). The stem cell connection of pituitary tumors. Front Endocrinol.

[25] Hass R (2020). Role of MSC in the tumor microenvironment. Cancers.

[26] Kudo-Saito C (2015). Cancer-associated mesenchymal stem cells aggravate tumor progression. Front Cell Dev Biol.

[27] Rovite V, Wolff-Sagi Y, Zaharenko L, Nikitina-Zake L, Grens E, Klovins J (2018). Genome database of the latvian population (LGDB): design, goals, and primary results. J Epidemiol.

[28] Ewels P, Magnusson M, Lundin S, Käller M (2016). MultiQC: summarize analysis results for multiple tools and samples in a single report. Bioinformatics.

[29] Chen S, Zhou Y, Chen Y, Gu J (2018). fastp: an ultra-fast all-in-one FASTQ preprocessor. Bioinformatics.

[30] Kopylova E, Noé L, Touzet H (2012). SortMeRNA: fast and accurate filtering of ribosomal RNAs in metatranscriptomic data. Bioinformatics.

[31] Patro R, Duggal G, Love MI, Irizarry RA, Kingsford C (2017). Salmon provides fast and bias-aware quantification of transcript expression. Nat Methods.

[32] R Core Team (2020). R: A Language and Environment for Statistical Computing.

[33] Love MI, Soneson C, Hickey PF, Johnson LK, Pierce NT, Shepherd L (2020). Tximeta: Reference sequence checksums for provenance identification in RNA-seq. PLoS Comput Biol.

[34] Love MI, Huber W, Anders S (2014). Moderated estimation of fold change and dispersion for RNA-seq data with DESeq2. Genome Biol.

[35] Ignatiadis N, Klaus B, Zaugg JB, Huber W (2016). Data-driven hypothesis weighting increases detection power in genome-scale multiple testing. Nat Methods.

[36] Zitovsky JP, Love MI (2020). Fast effect size shrinkage software for beta-binomial models of allelic imbalance. F1000Res.

[37] Kolde R. Pheatmap: Pretty Heatmaps. CRAN Repository. 2019.

[38] Blighe K, Rana S, Lewis M. EnhancedVolcano: Publication-ready volcano plots with enhanced colouring and labeling. 2022.

[39] Wickham H (2016). ggplot2: Elegant Graphics for Data Analysis.

[40] Szklarczyk D, Gable AL, Nastou KC, Lyon D, Kirsch R, Pyysalo S (2021). The STRING database in 2021: customizable protein–protein networks, and functional characterization of user-uploaded gene/measurement sets. Nucleic Acids Res.

[41] Strimmer K (2008). fdrtool: a versatile R package for estimating local and tail area-based false discovery rates. Bioinformatics.

[42] Xu M, Shorts-Cary L, Knox AJ, Kleinsmidt-DeMasters B, Lillehei K, Wierman ME (2009). Epidermal growth factor receptor pathway substrate 8 is overexpressed in human pituitary tumors: role in proliferation and survival. Endocrinology.

[43] LeRiche VK, Asa SL, Ezzat S (1996). Epidermal growth factor and its receptor (EGF-R) in human pituitary adenomas: EGF-R correlates with tumor aggressiveness. J Clin Endocrinol Metab.

[44] Araki T, Liu X, Kameda H, Tone Y, Fukuoka H, Tone M (2017). EGFR induces E2F1-mediated corticotroph tumorigenesis. J Endocr Soc.

[45] Rai A, Das L, Mukherjee KK, Dhandapani S, Tripathi M, Ahuja CK (2021). Phosphorylated EGFR (pEGFR T693) as a novel predictor of recurrence in non-functioning pituitary adenomas. Front Endocrinol.

[46] Lebrun J-J (2009). Activin, TGF-beta and menin in pituitary tumorigenesis. Adv Exp Med Biol.

[47] Picech F, Sosa LD, Perez PA, Cecenarro L, Oms SR, Coca HA (2021). TGF-β1/Smad2/3 signaling pathway modulates octreotide antisecretory and antiproliferative effects in pituitary somatotroph tumor cells. J Cell Physiol.

[48] Sjöstedt E, Kolnes AJ, Olarescu NC, Mitsios N, Hikmet F, Sivertsson Å (2020). TGFBR3L-an uncharacterised pituitary specific membrane protein detected in the gonadotroph cells in non-neoplastic and tumour tissue. Cancers.

[49] Øystese KAB, Berg JP, Normann KR, Zucknick M, Casar-Borota O, Bollerslev J (2018). The role of E and N-cadherin in the postoperative course of gonadotroph pituitary tumours. Endocrine.

[50] Venegas-Moreno E, Flores-Martinez A, Dios E, Vazquez-Borrego MC, Ibañez-Costa A, Madrazo-Atutxa A (2019). E-cadherin expression is associated with somatostatin analogue response in acromegaly. J Cell Mol Med.

[51] Falch CM, Sundaram AYM, Øystese KA, Normann KR, Lekva T, Silamikelis I (2018). Gene expression profiling of fast- and slow-growing non-functioning gonadotroph pituitary adenomas. Eur J Endocrinol.

[52] Fougner SL, Lekva T, Borota OC, Hald JK, Bollerslev J, Berg JP (2010). The expression of E-cadherin in somatotroph pituitary adenomas is related to tumor size, invasiveness, and somatostatin analog response. J Clin Endocrinol Metab.

[53] Ezzat S, Zheng L, Asa SL (2004). Pituitary tumor-derived fibroblast growth factor receptor 4 isoform disrupts neural cell-adhesion molecule/N-Cadherin signaling to diminish cell adhesiveness: a mechanism underlying pituitary neoplasia. Mol Endocrinol.

[54] Ezzat S, Zheng L, Winer D, Asa SL (2006). Targeting N-cadherin through fibroblast growth factor receptor-4: distinct pathogenetic and therapeutic implications. Mol Endocrinol.

[55] Qian ZR, Sano T, Yoshimoto K, Asa SL, Yamada S, Mizusawa N (2007). Tumor-specific downregulation and methylation of the CDH13 (H-cadherin) and CDH1 (E-cadherin) genes correlate with aggressiveness of human pituitary adenomas. Mod Pathol.

[56] Menon SS, Guruvayoorappan C, Sakthivel KM, Rasmi RR (2019). Ki-67 protein as a tumour proliferation marker. Clin Chim Acta.

[57] de Araújo LJT, Lerario AM, de Castro M, Martins CS, Bronstein MD, Machado MC (2017). Transcriptome analysis showed a differential signature between invasive and non-invasive corticotrophinomas. Front Endocrinol.

[58] Ghatnatti V, Vastrad B, Patil S, Vastrad C, Kotturshetti I (2021). Identification of potential and novel target genes in pituitary prolactinoma by bioinformatics analysis. AIMS Neurosci.

[59] Song Z-J, Reitman ZJ, Ma Z-Y, Chen J-H, Zhang Q-L, Shou X-F (2016). The genome-wide mutational landscape of pituitary adenomas. Cell Res.

[60] Välimäki N, Demir H, Pitkänen E, Kaasinen E, Karppinen A, Kivipelto L (2015). Whole-genome sequencing of growth hormone (GH)-secreting pituitary adenomas. J Clin Endocrinol Metab.

[61] Lanner JT, Georgiou DK, Joshi AD, Hamilton SL (2010). Ryanodine receptors: structure, expression, molecular details, and function in calcium release. Cold Spring Harb Perspect Biol.

[62] Kushnir A, Wajsberg B, Marks AR (2018). Ryanodine receptor dysfunction in human disorders. Biochim Biophys Acta.

[63] Ronchi CL, Peverelli E, Herterich S, Weigand I, Mantovani G, Schwarzmayr T (2016). Landscape of somatic mutations in sporadic GH-secreting pituitary adenomas. Eur J Endocrinol.

[64] Peverelli E, Mantovani G, Lania AG, Spada A (2014). cAMP in the pituitary: an old messenger for multiple signals. J Mol Endocrinol.

[65] Sundaresan S, Weiss J, Bauer-Dantoin AC, Jameson JL (1997). Expression of ryanodine receptors in the pituitary gland: evidence for a role in gonadotropin-releasing hormone signaling. Endocrinology.

[66] Fougner SL, Borota OC, Berg JP, Hald JK, Ramm-Pettersen J, Bollerslev J (2008). The clinical response to somatostatin analogues in acromegaly correlates to the somatostatin receptor subtype 2a protein expression of the adenoma. Clin Endocrinol.

[67] Franck SE, Gatto F, van der Lely AJ, Janssen JAMJL, Dallenga AHG, Nagtegaal AP (2017). Somatostatin receptor expression in GH-secreting pituitary adenomas treated with long-acting somatostatin analogues in combination with pegvisomant. Neuroendocrinology.

[68] Hu J, Yin H, Li B, Yang H (2019). <p>Identification of transcriptional metabolic dysregulation in subtypes of pituitary adenoma by integrated bioinformatics analysis</p>. Diabetes Metab Syndr Obes.

[69] Moreno CS, Evans C-O, Zhan X, Okor M, Desiderio DM, Oyesiku NM (2005). Novel molecular signaling and classification of human clinically nonfunctional pituitary adenomas identified by gene expression profiling and proteomic analyses. Cancer Res.

[70] Mantovani G, Treppiedi D, Giardino E, Catalano R, Mangili F, Vercesi P (2019). Cytoskeleton actin-binding proteins in clinical behavior of pituitary tumors. Endocr Relat Cancer.

[71] Paragliola RM, Corsello SM, Salvatori R (2017). Somatostatin receptor ligands in acromegaly: clinical response and factors predicting resistance. Pituitary.

[72] Wang X, Xie J, Proud C (2017). Eukaryotic elongation factor 2 kinase (eEF2K) in cancer. Cancers.

[73] Thul PJ, Lindskog C (2018). The human protein atlas: a spatial map of the human proteome. Protein Sci.

[74] Timaxian C, Raymond-Letron I, Bouclier C, Gulliver L, le Corre L, Chébli K (2020). The health status alters the pituitary function and reproduction of mice in a *Cxcr2* -dependent manner. Life Sci Alliance.

[75] Tofrizal A, Fujiwara K, Azuma M, Kikuchi M, Jindatip D, Yashiro T (2017). Tissue inhibitors of metalloproteinase-expressing cells in human anterior pituitary and pituitary adenoma. Med Mol Morphol.

[76] Tofrizal A, Fujiwara K, Yashiro T, Yamada S (2016). Alterations of collagen-producing cells in human pituitary adenomas. Med Mol Morphol.

[77] Long R, Liu Z, Li J, Yu H (2019). COL6A6 interacted with P4HA3 to suppress the growth and metastasis of pituitary adenoma via blocking PI3K-Akt pathway. Aging.

[78] Abe T, Ludecke D (2001). Effects of preoperative octreotide treatment on different subtypes of 90 GH-secreting pituitary adenomas and outcome in one surgical centre. Eur J Endocrinol.

[79] Carlsen SM, Lund-Johansen M, Schreiner T, Aanderud S, Johannesen Ø, Svartberg J (2008). Preoperative octreotide treatment in newly diagnosed acromegalic patients with macroadenomas increases cure short-term postoperative rates: a prospective. Randomized Trial J Clin Endocrinol Metab.

[80] Leek JT, Scharpf RB, Bravo HC, Simcha D, Langmead B, Johnson WE (2010). Tackling the widespread and critical impact of batch effects in high-throughput data. Nat Rev Genet.

[81] Nishioka H, Inoshita N (2018). New WHO classification of pituitary adenomas (4th edition): assessment of pituitary transcription factors and the prognostic histological factors. Brain Tumor Pathol.

