# Supplementary material for: Transcriptome of GH-producing pituitary neuroendocrine tumours and models are significantly affected by somatostatin analogues
Source: Cancer Cell Int. 2023 Feb 11;23:25. doi: 10.1186/s12935-023-02863-4 (PMC9922463; doi:10.1186/s12935-023-02863-4)
Supplement: Supplementary file 3 — Additional file 3: Figure S1. Variance stabilization transformed heatmap of mean normalized gene level counts for DEGs of the sompatotroph and non-secreting PitNET type comparison. Pink fields in the “Subtype” column row, represent the non-secreting group samples, green fields represent the non-secreting group samples. Figure S2. Summarization of the categories in enrichment analysis results for the PitNET non-secreting and somatotroph type comparison. Each bar represents an enrichment category with the corresponding occurance in enrichment results. Figure S3. A STRING-db network for the DEG proteins of somatotroph and non-secreting PitNET type comparison. Line thickness indicates confidence of protein interaction. Disconnected nodes were hidden. Text mining disabled as interaction type. Figure S4. ASTRING -db network for the DEG proteins of somatotroph and non-secreting PitNET type comparison. Line colour indicates protein interaction type and line count indicates number of interactions. Disconnected nodes were hidden. Text mining disabled as interaction type. Figure S5. ASTRING -db network for the DEG proteins of SSA treated/untreted somatotroph PitNET comparison. Line thickness indicates interaction confidence. Disconnected nodes were hidden. Text mining disabled as interaction type. Figure S6. A STRING-db network for the DEG proteins of SSA treated/untreted somatotroph PitNET comparison. Line colour indicated interaction type and line count indicates number of interactions. Disconnected nodes were hidden. Text mining disabled as interaction type. Figure S7. Variance stabilization transformed heatmap of mean normalized gene level counts for DEGs of the octreotide incubated pituispheres cell and pituisphere control comparsion at 4 hours. Bright green (middle) represents samples from the octreotide 4h incubation period, darker green represents the unincubated pituisphere control samples. Figure S8. Variance stabilization transformed heatmap of mean normalized gene [file 12935_2023_2863_MOESM3_ESM.pdf]

Additional file figures

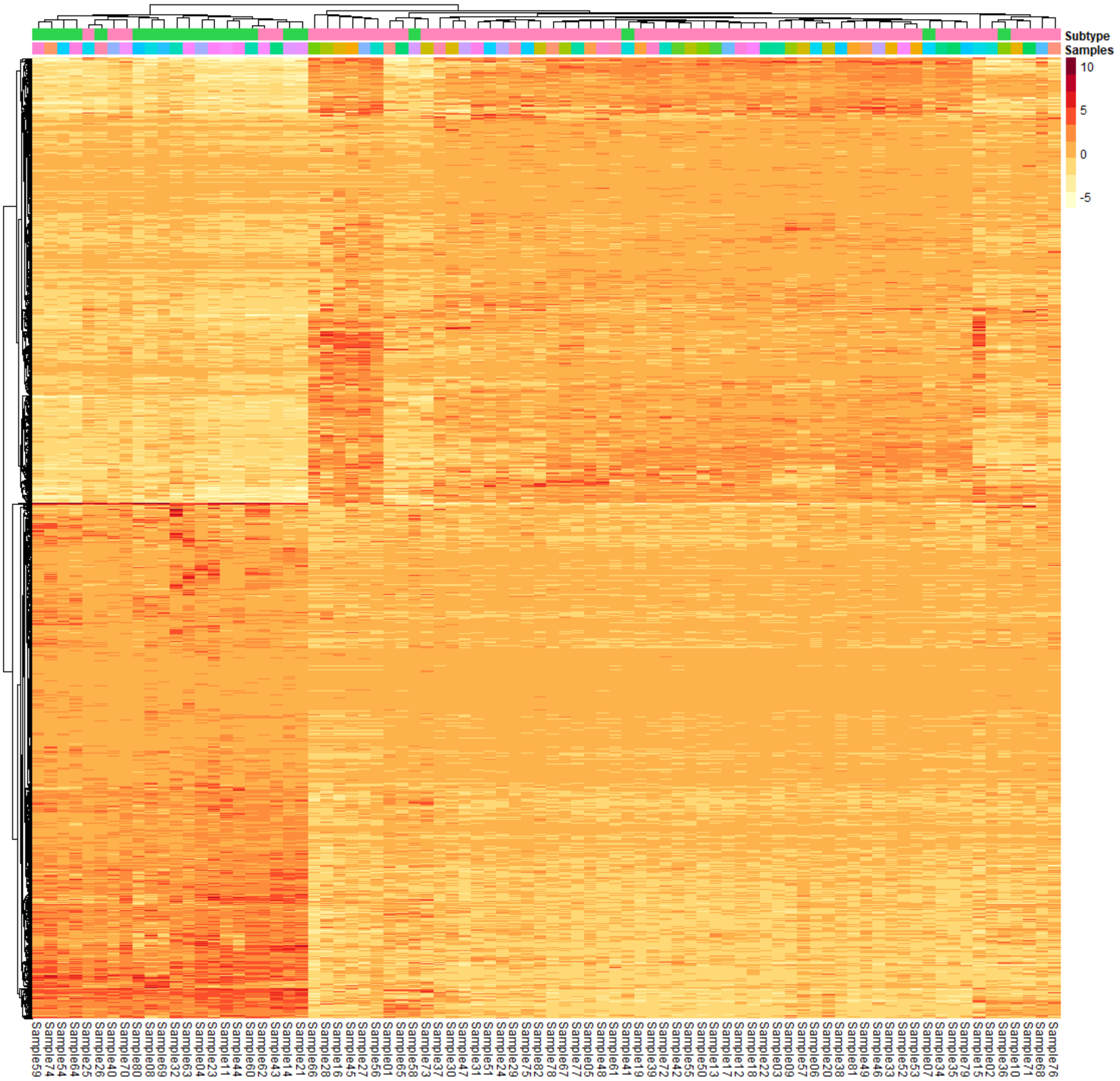

Figure S1. Variance stabilization transformed heatmap of mean normalized gene level counts for DEGs of the sompatotroph and non-secreting PitNET type comparison. Pink fields in the “Subtype” column row, represent the non-secreting group samples, green fields represent the non-secreting group samples.

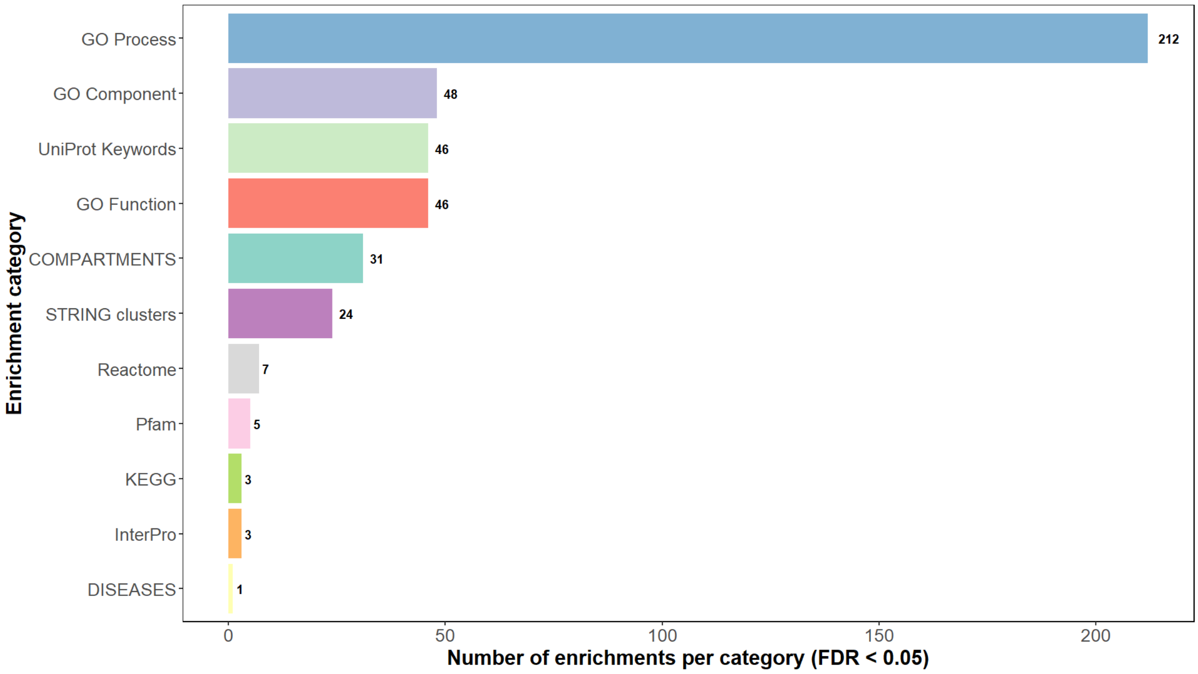

Figure S2. Summarization of the categories in enrichment analysis results for the PitNET non-secreting and somatotroph type comparison. Each bar represents an enrichment category with the corresponding occurance in enrichment results.

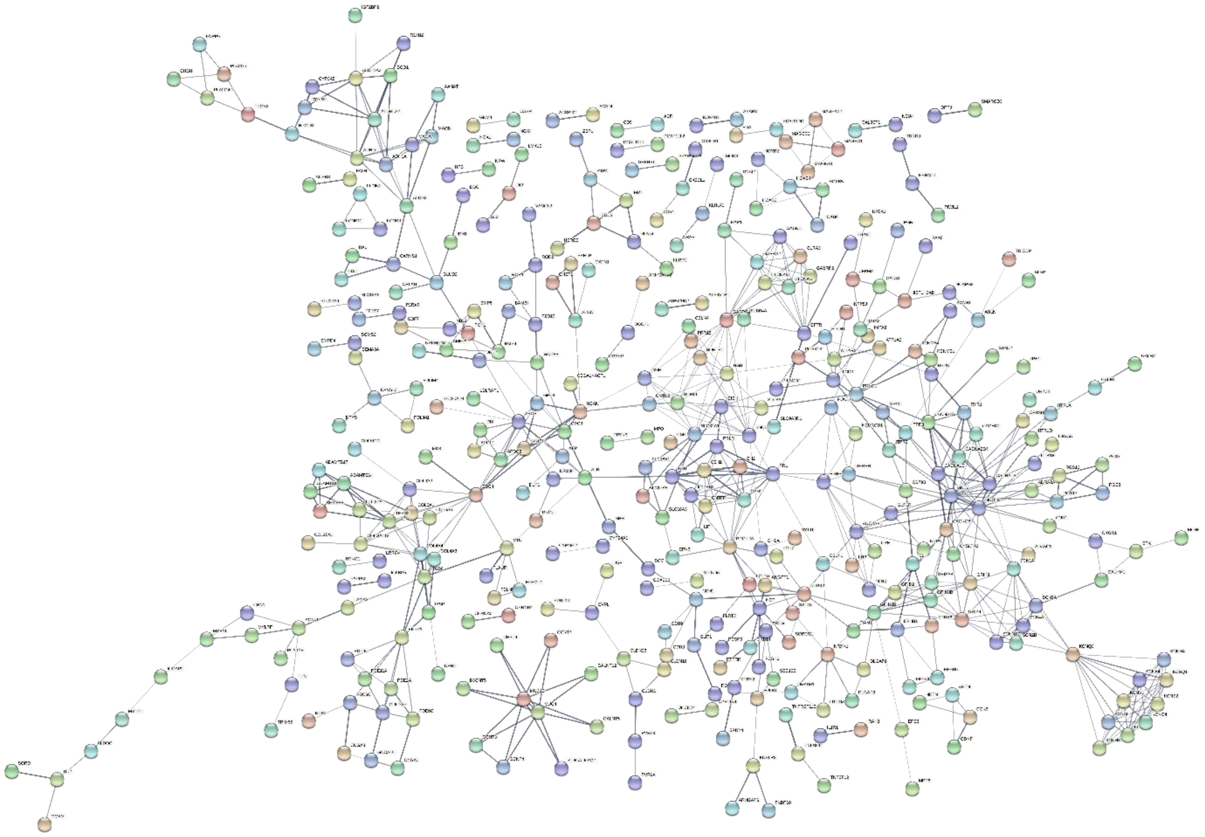

Figure S3. A STRING-db network for the DEG proteins of somatotroph and non - secreting PitNET type comparison. Line thickness indicates confidence of protein interaction. Disconnected nodes were hidden. Text mining disabled as interaction type.

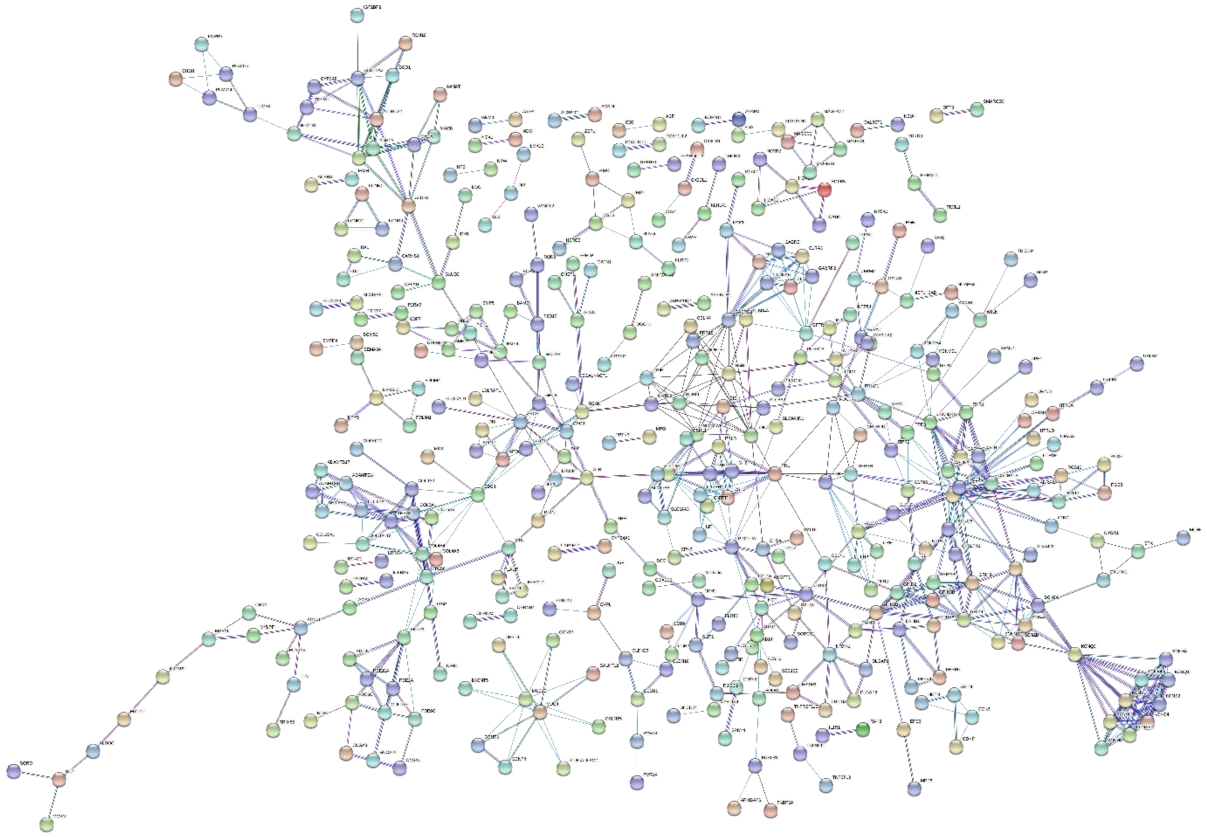

Figure S4. A STRING db network-for the DEG proteins of somatotroph and non-secreting PitNET type comparison. Line colour indicates protein interaction type and line count indicates number of interactions. Disconnected nodes were hidden. Text mining disabled as interaction type.

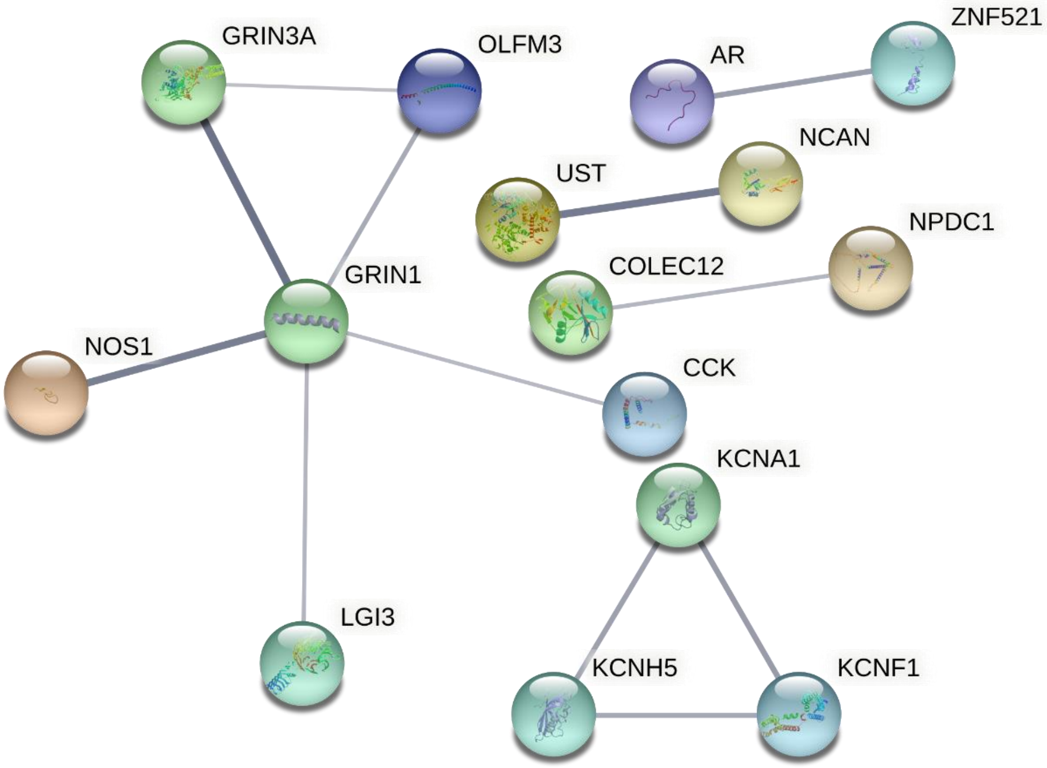

Figure S5 A STRING-db network for the DEG proteins of SSA treated/untreted somatotroph PitNET comparison. Line thickness indicates interaction confidence. Disconnected nodes were hidden. Text mining disabled as interaction type.

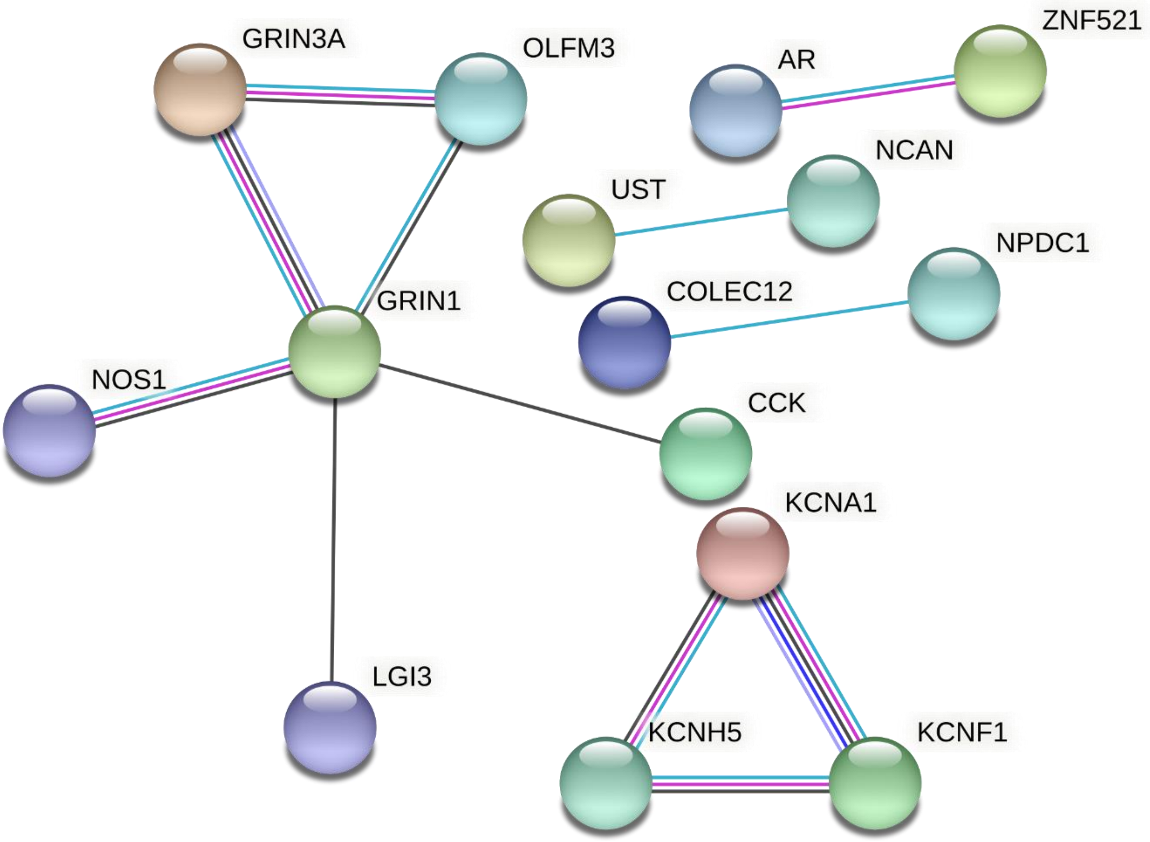

Figure S6. A STRING-db network for the DEG proteins of SSA treated/untreted somatotroph PitNET comparison. Line colour indicated interaction type and line count indicates number of interactions. Disconnected nodes were hidden. Text mining disabled as interaction type.

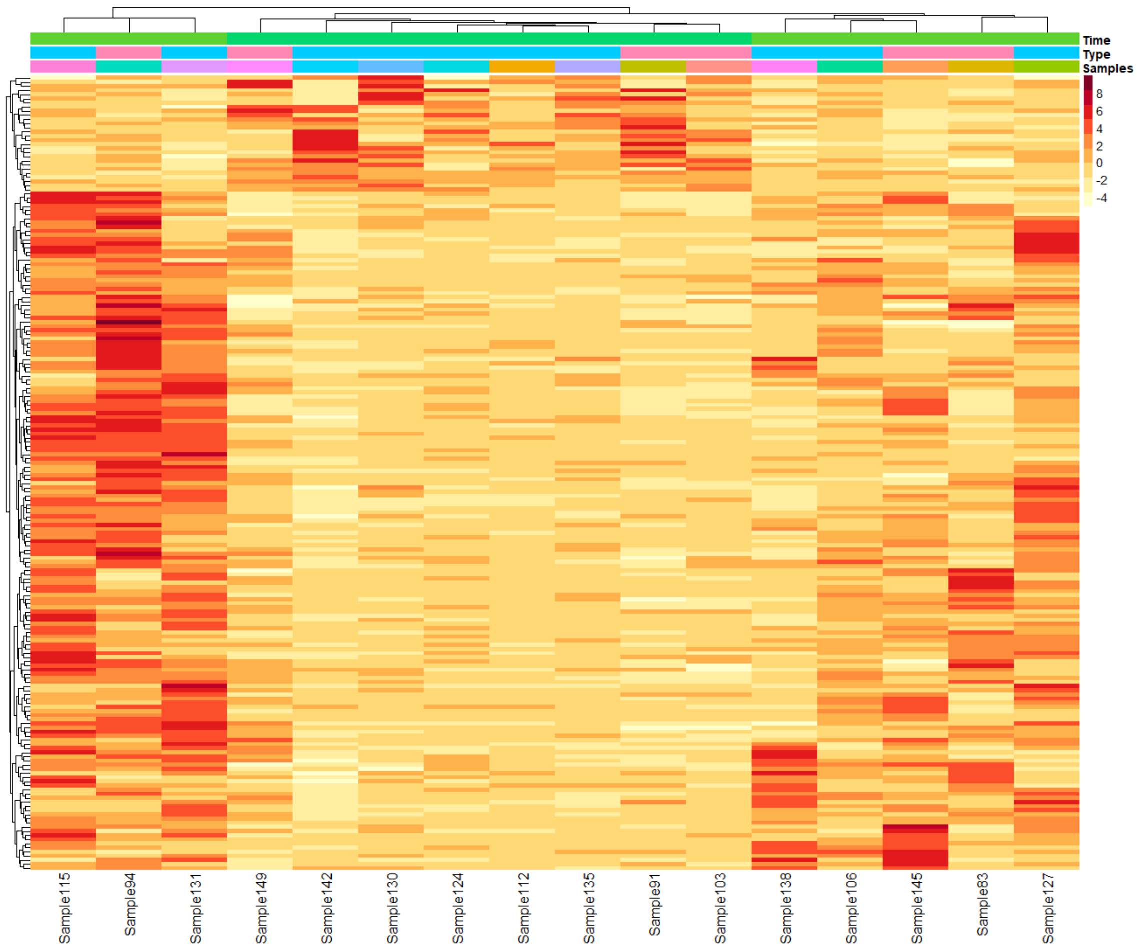

Figure S7. Variance stabilization transformed heatmap of mean normalized gene level counts for DEGs of the octreotide incubated pituispheres cell and pituisphere control comparsion at 4 hours. Bright green (middle) represents samples from the octreotide 4h incubation period, darker green represents the unincubated pituisphere control samples.

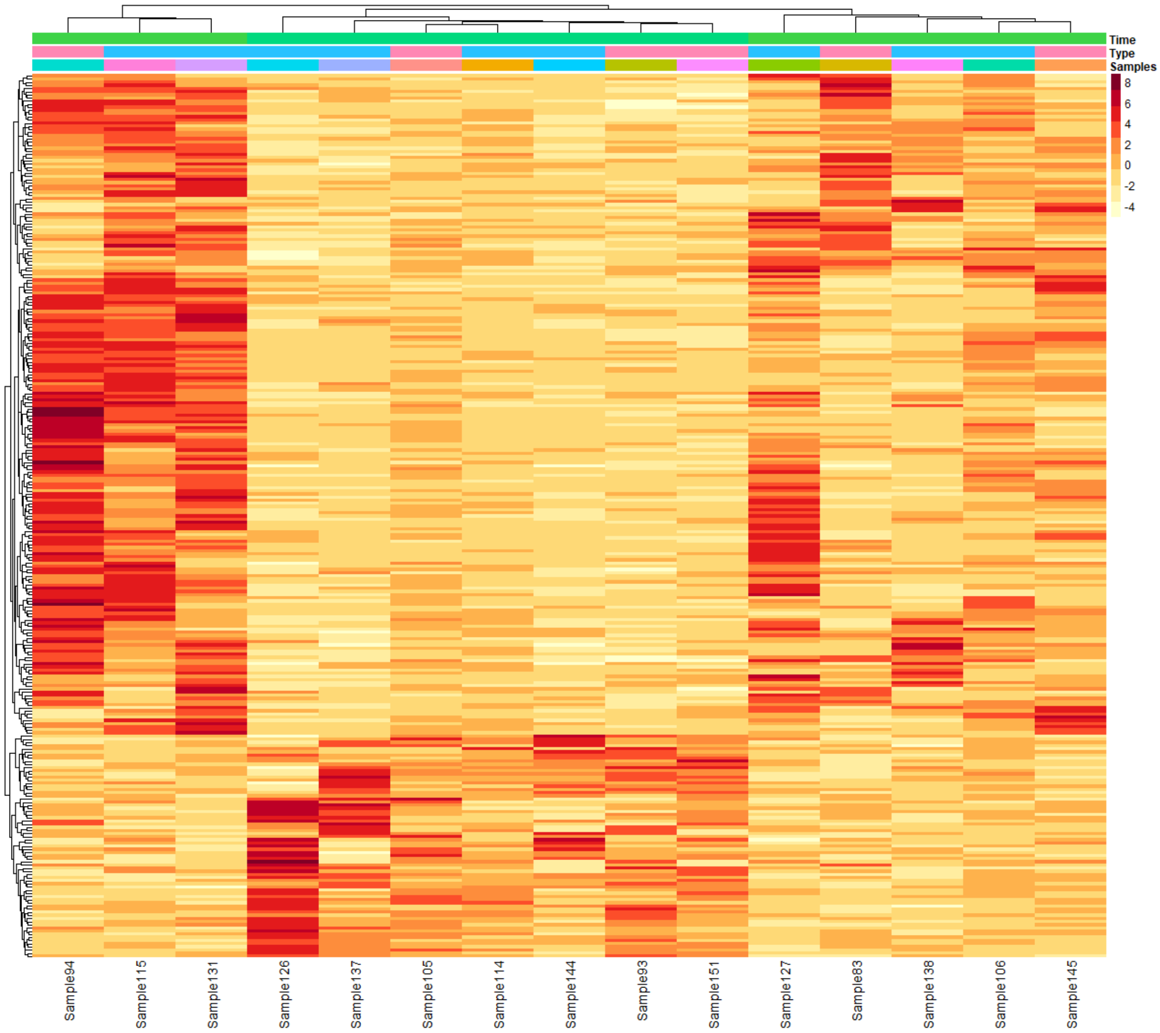

Figure S8. Variance stabilization transformed heatmap of mean normalized gene level counts for DEGs of the octreotide incubated pituispheres cell and pituisphere control comparsion at 8 hours. Dark green represents the unincubated pituisphere control samples, bright green represents the octreotide incubated pituispheres at 8 hours.

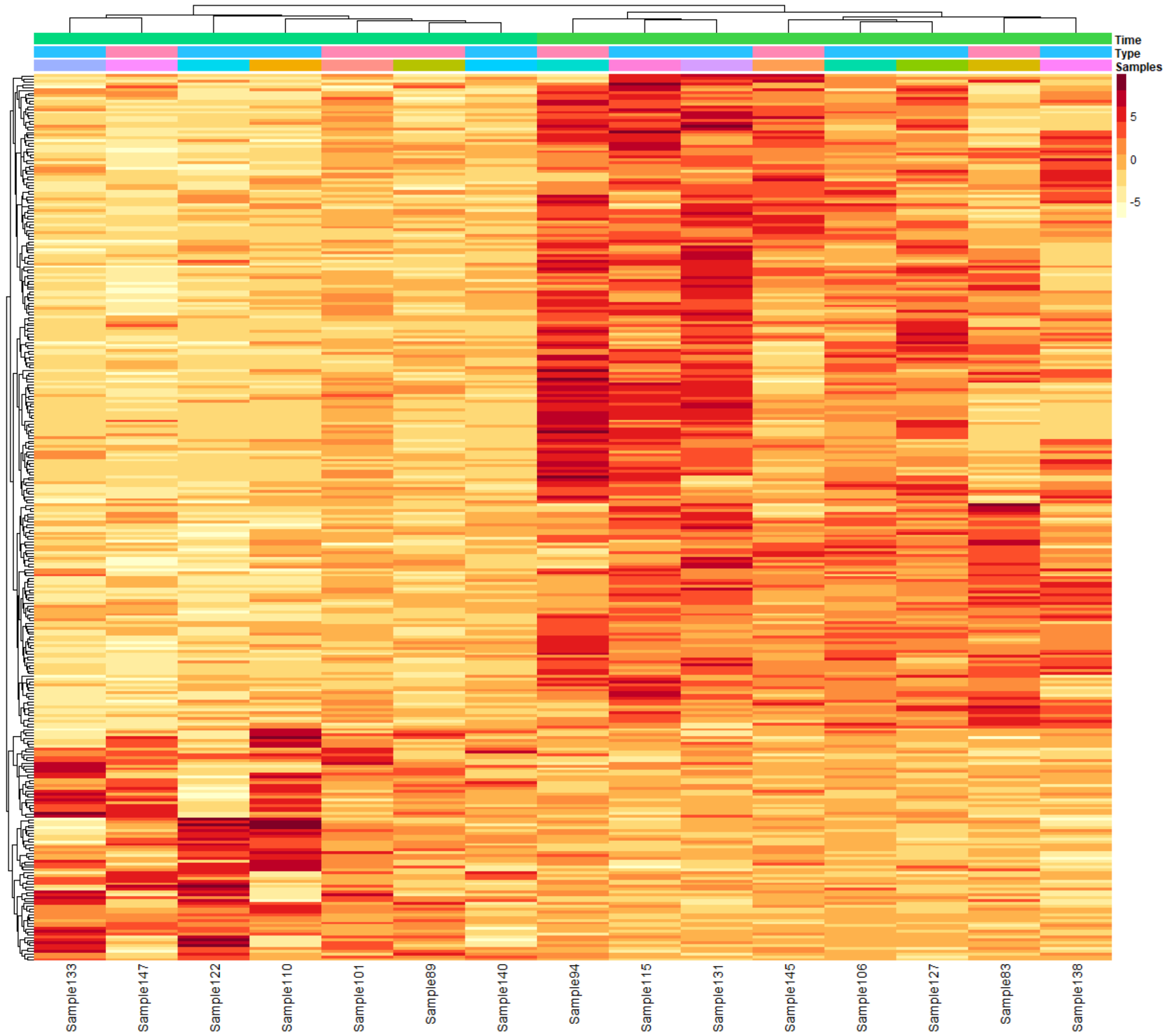

Figure S9. Variance stabilization transformed heatmap of mean normalized gene level counts for DEGs of the octreotide incubated pituispheres cell and pituisphere control comparsion at 24 hours. Dark green represents the unincubated pituisphere control samples, bright green represents the octreotide incubated pituispheres at 24 hours.

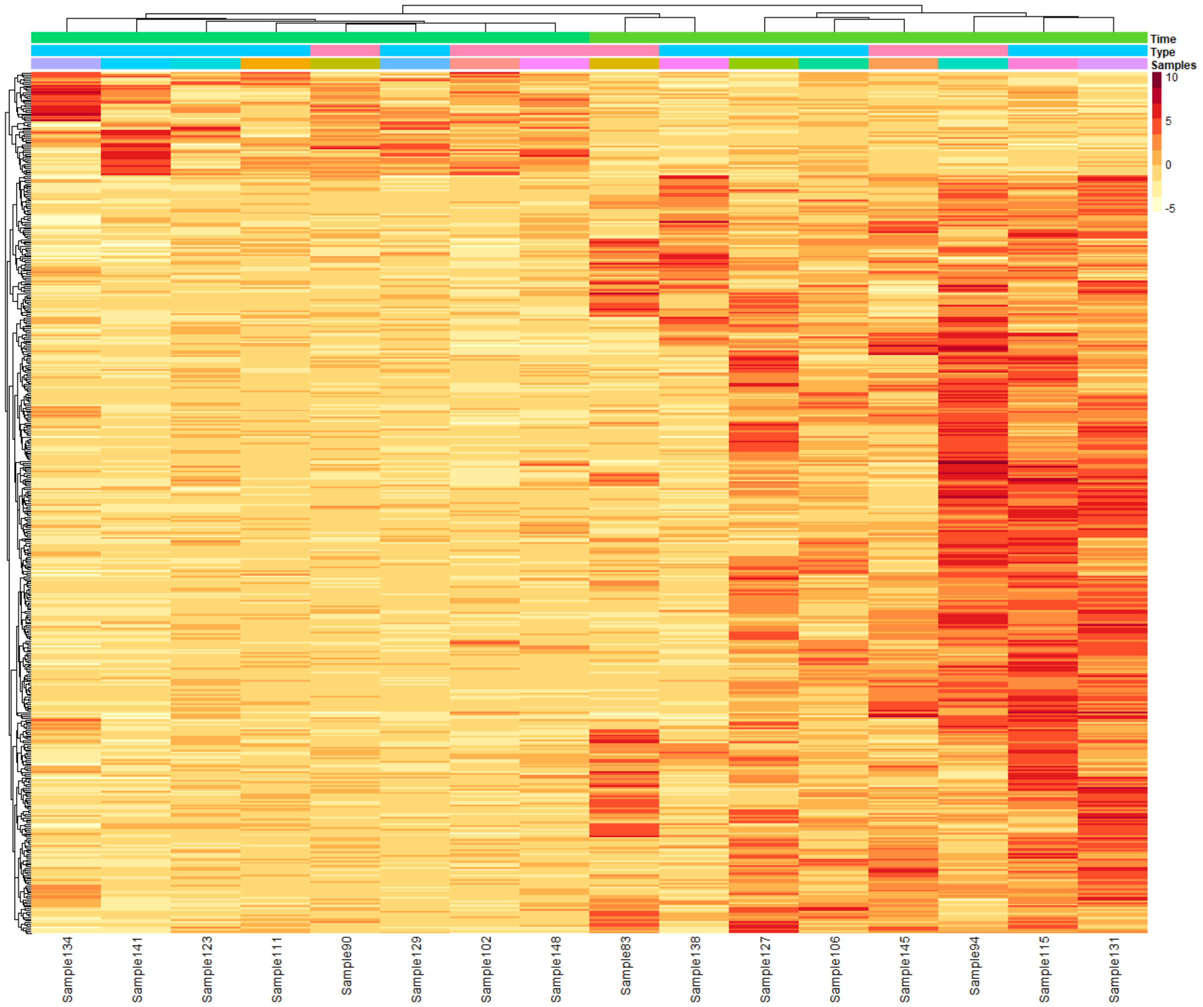

Figure S10. Variance stabilization transformed heatmap of mean normalized gene level counts for DEGs of the octreotide incubated pituispheres cell and pituisphere control comparsion at 48 hours. Dark green represents the unincubated pituisphere control samples, bright green represents the octreotide incubated pituispheres at 48 hours.

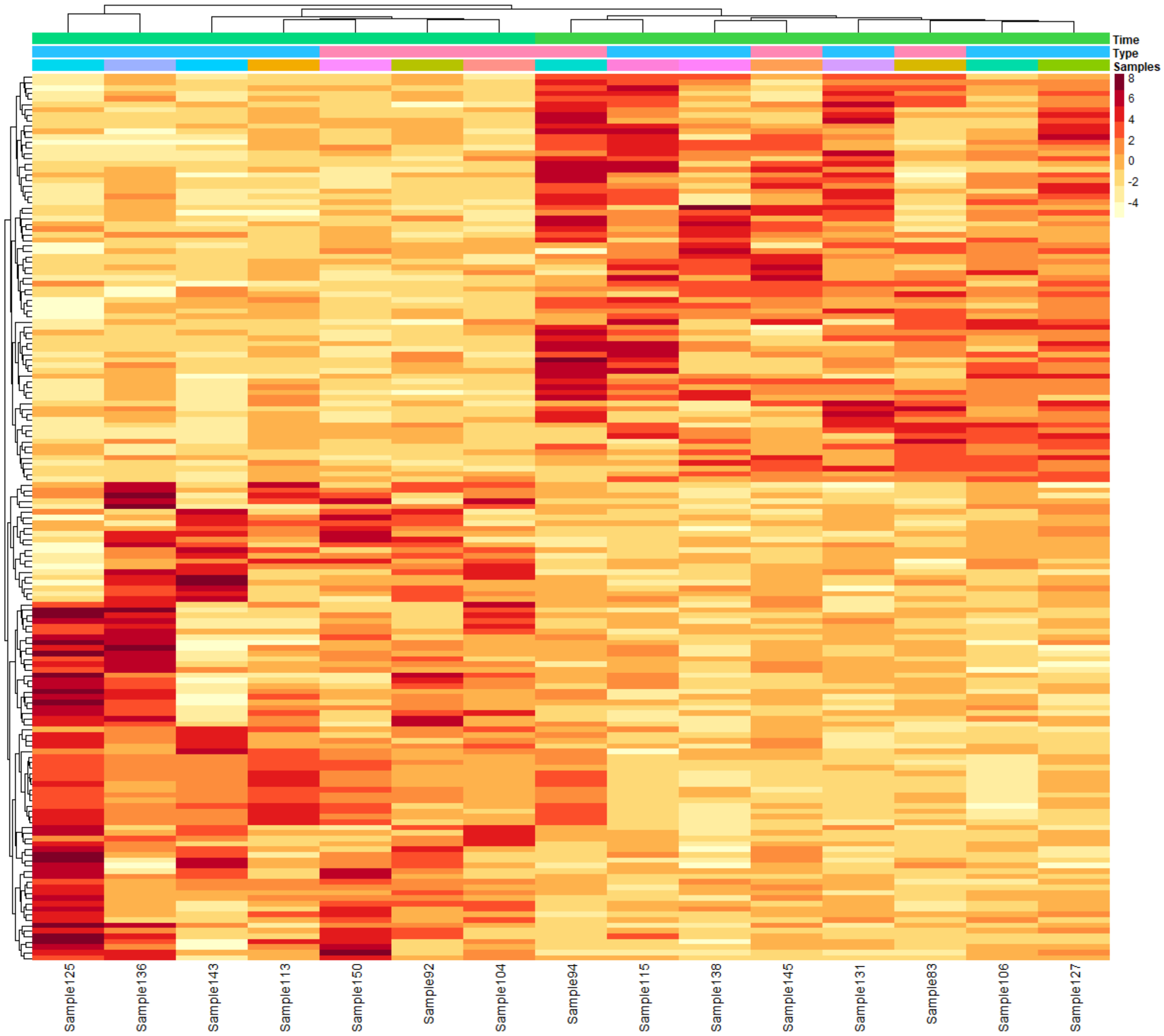

Figure S11. Variance stabilization transformed heatmap of mean normalized gene level counts for DEGs of the octreotide incubated pituispheres cell and pituisphere control comparsion at 72 hours. Dark green represents the unincubated pituisphere control samples, bright green represents the octreotide incubated pituispheres at 72 hours.

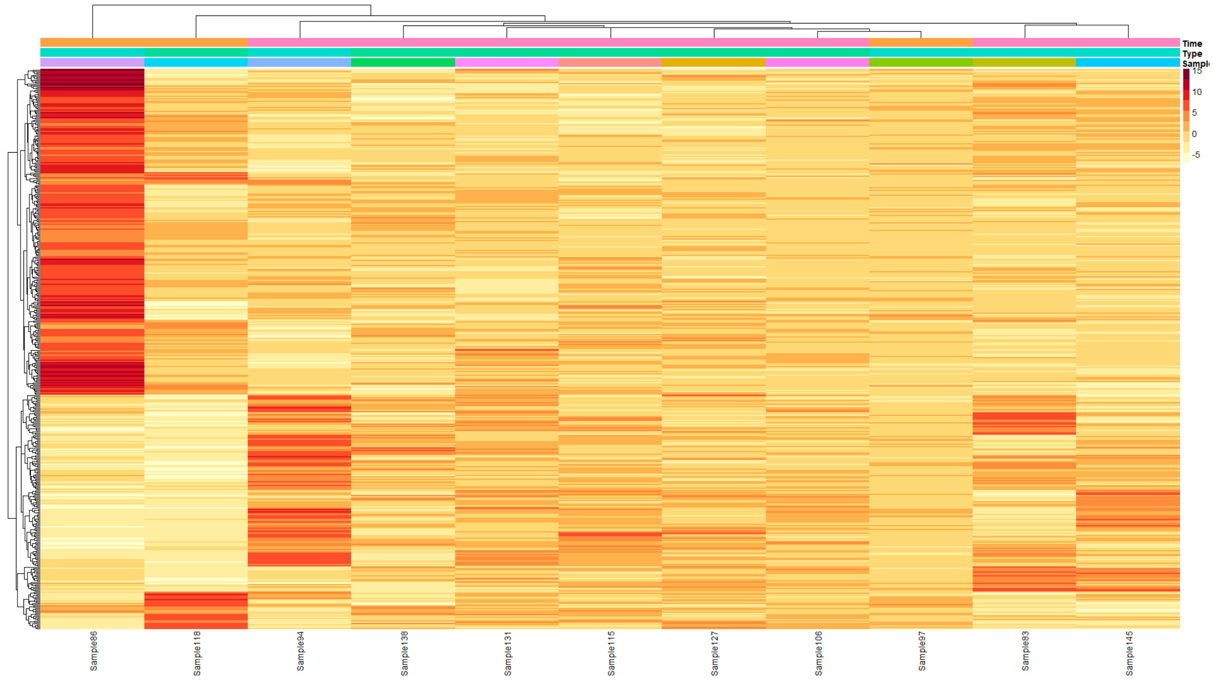

Figure S12. Variance stabilization transformed heatmap of mean normalized gene level counts for DEGs of the cabergoline incubated pituispheres cell and pituisphere control comparsion at 4 hours. Pink represents the unincubated pituisphere control samples, orange represents the octreotide incubated pituispheres at 4 hours.

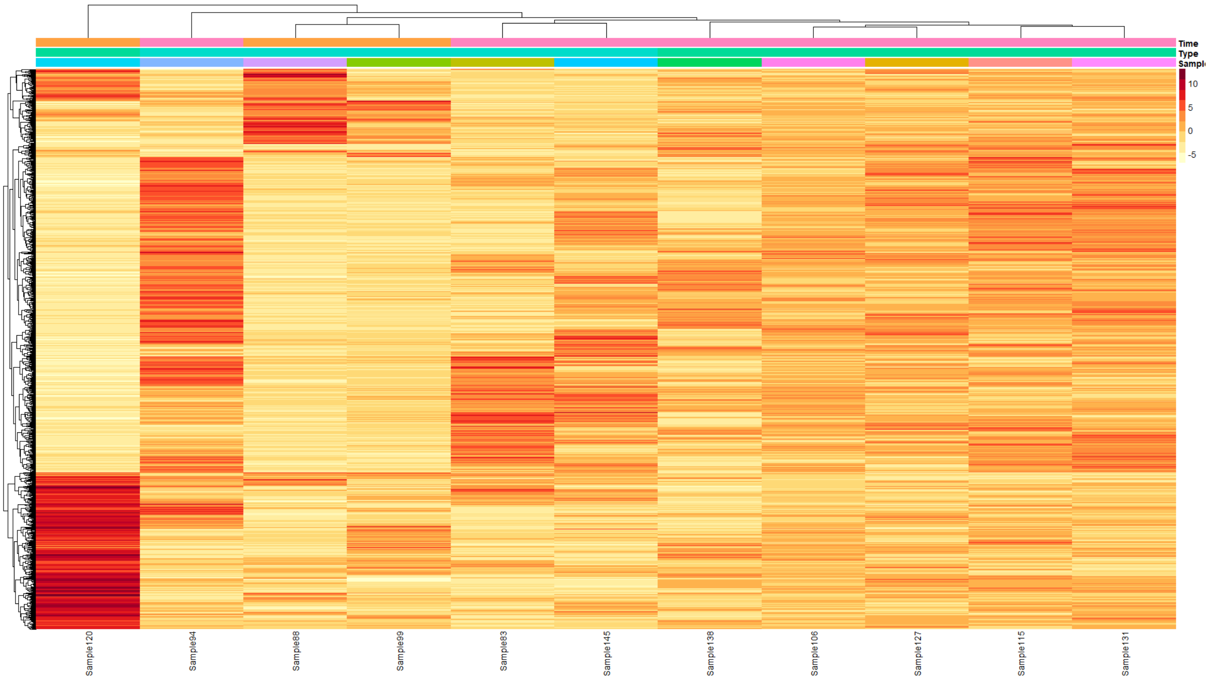

Figure S13. Variance stabilization transformed heatmap of mean normalized gene level counts for DEGs of the cabergoline incubated pituispheres cell and pituisphere control comparsion at 8 hours. Pink represents the unincubated pituisphere control samples, orange represents the octreotide incubated pituispheres at 8 hours.

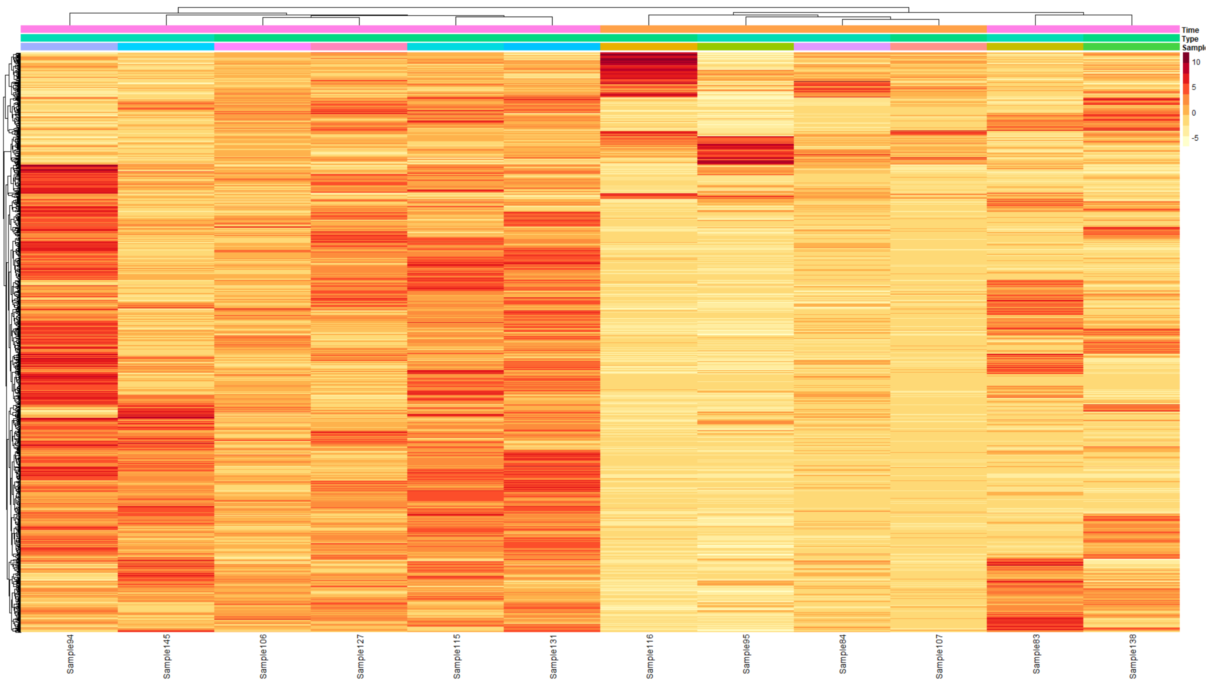

Figure S14. Variance stabilization transformed heatmap of mean normalized gene level counts for DEGs of the cabergoline incubated pituispheres cell and pituisphere control comparsion at 24 hours. Pink represents the unincubated pituisphere control samples, orange represents the octreotide incubated pituispheres at 24 hours.

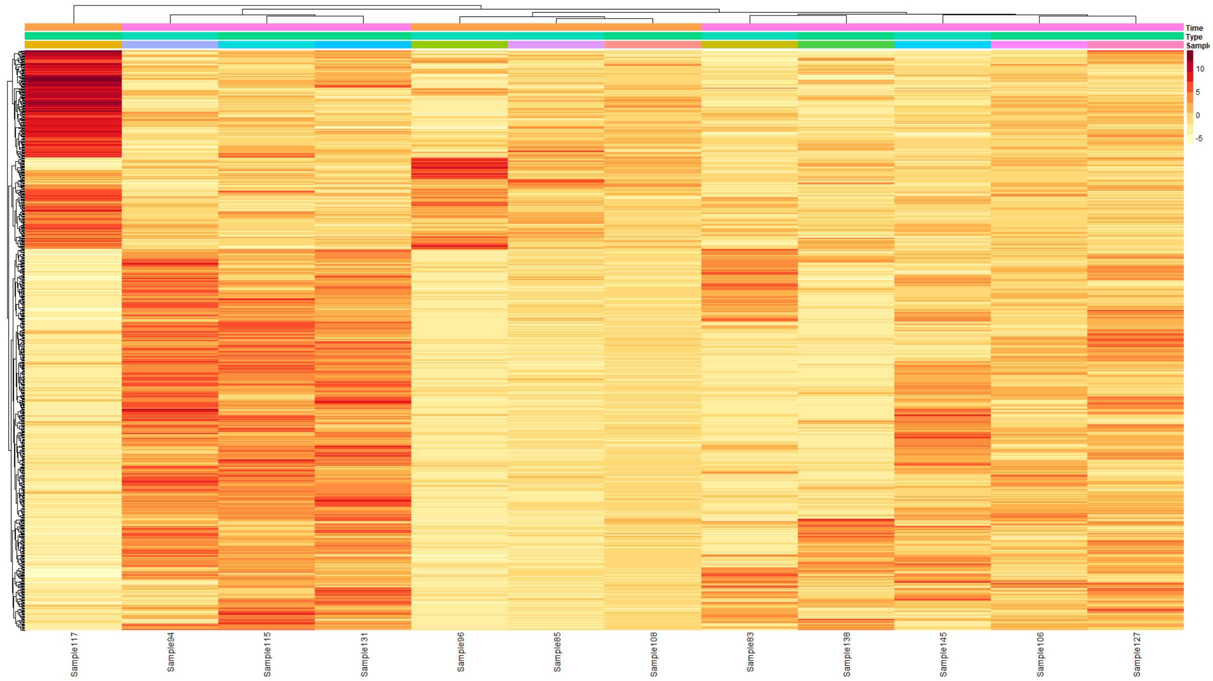

Figure S15. Variance stabilization transformed heatmap of mean normalized gene level counts for DEGs of the cabergoline incubated pituispheres cell and pituisphere control comparsion at 48 hours. Pink represents the unincubated pituisphere control samples, orange represents the octreotide incubated pituispheres at 48 hours.

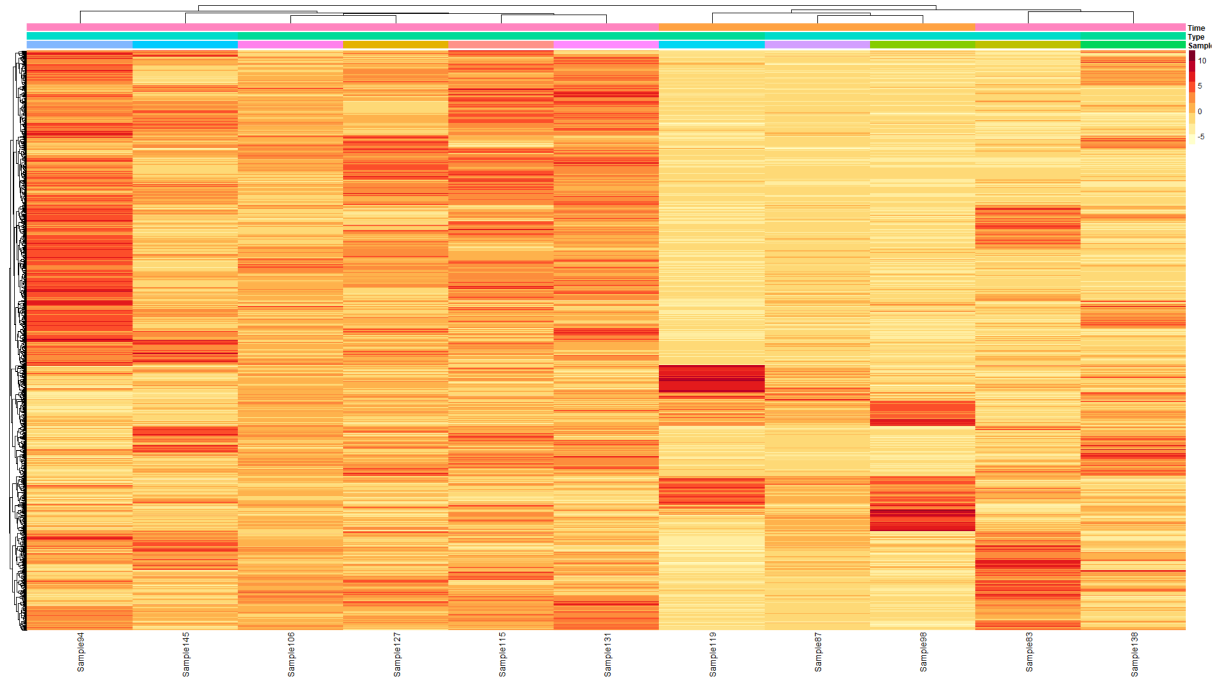

Figure S16. Variance stabilization transformed heatmap of mean normalized gene level counts for DEGs of the cabergoline incubated pituispheres cell and pituisphere control comparsion at 72 hours. Pink represents the unincubated pituisphere control samples, orange represents the octreotide incubated pituispheres at 72 hours.

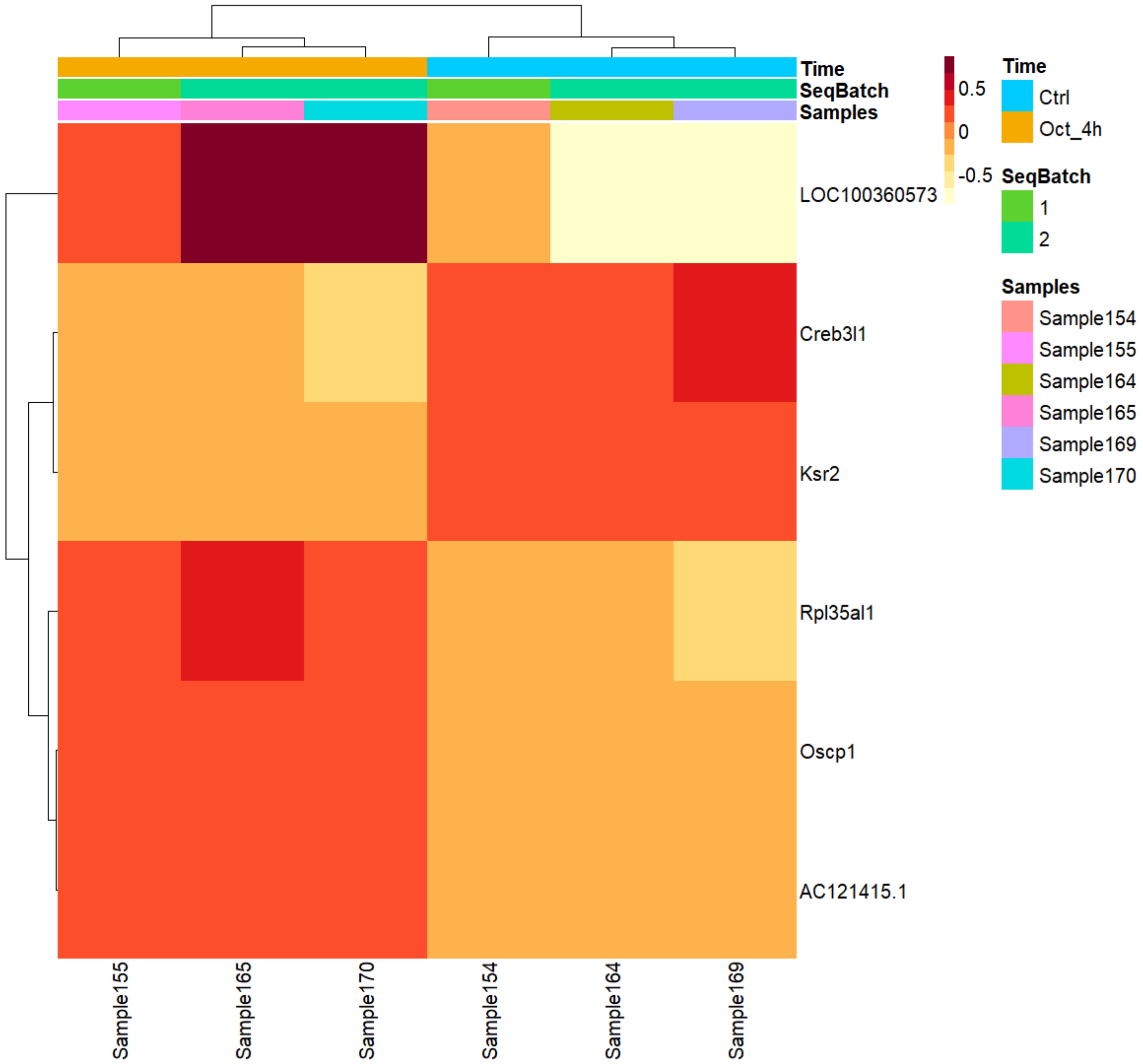

Figure S17. Variance stabilization transformed heatmap of mean normalized gene level counts for DEGs of the octreotide incubated GH3 cell and GH3 control comparsion at 4 hours. Light blue fields in the “Time” column row, represent the unincubated control samples, orange fields represent the GH3 samples incubated with octreotide at 4 hours.

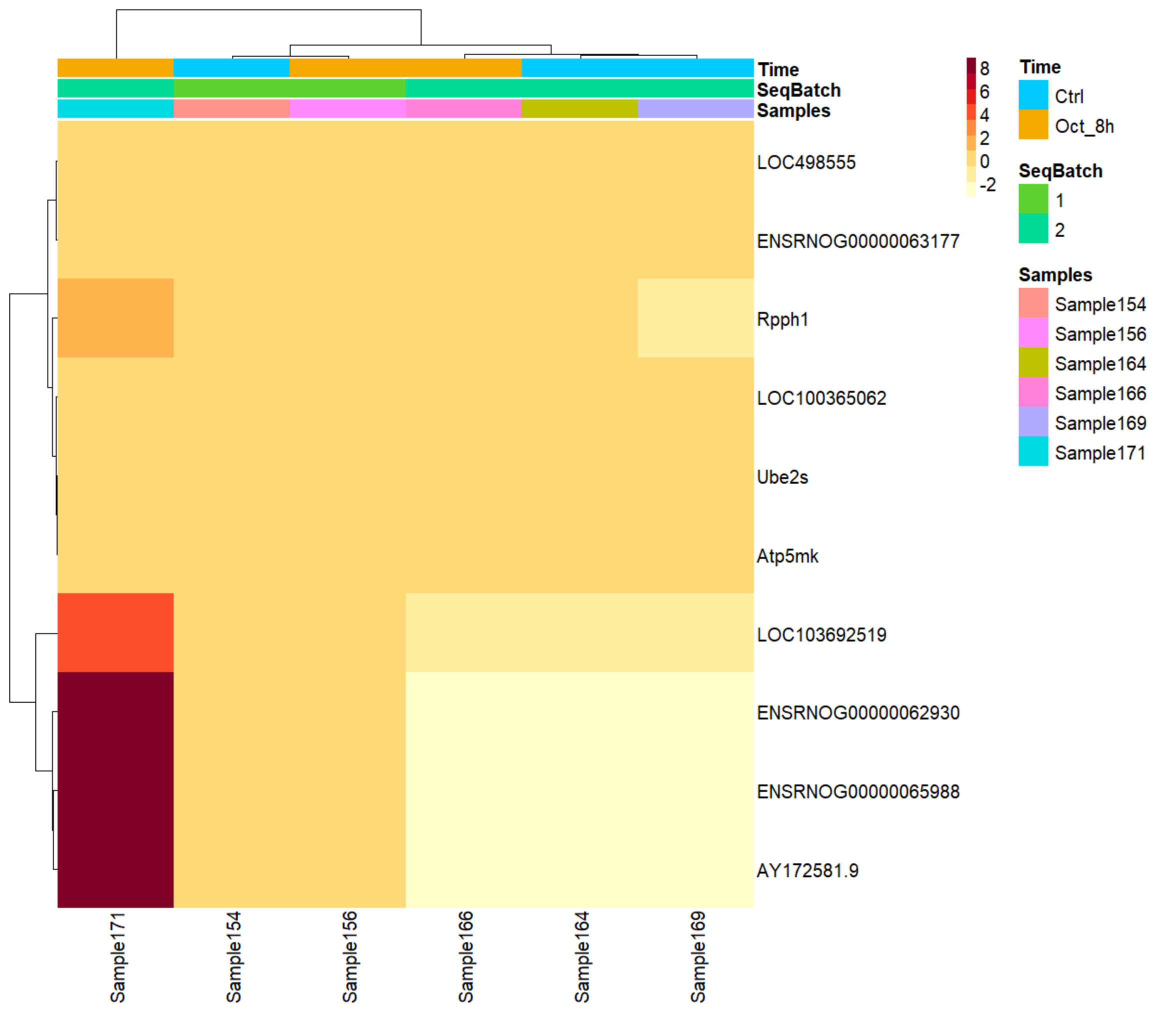

Figure S18. Variance stabilization transformed heatmap of mean normalized gene level counts for DEGs of the octreotide incubated GH3 cell and GH3 control comparsion at 8 hours. Light blue fields in the “Time” column row, represent the unincubated control samples, orange fields represent the GH3 samples incubated with octreotide at 8 hours.

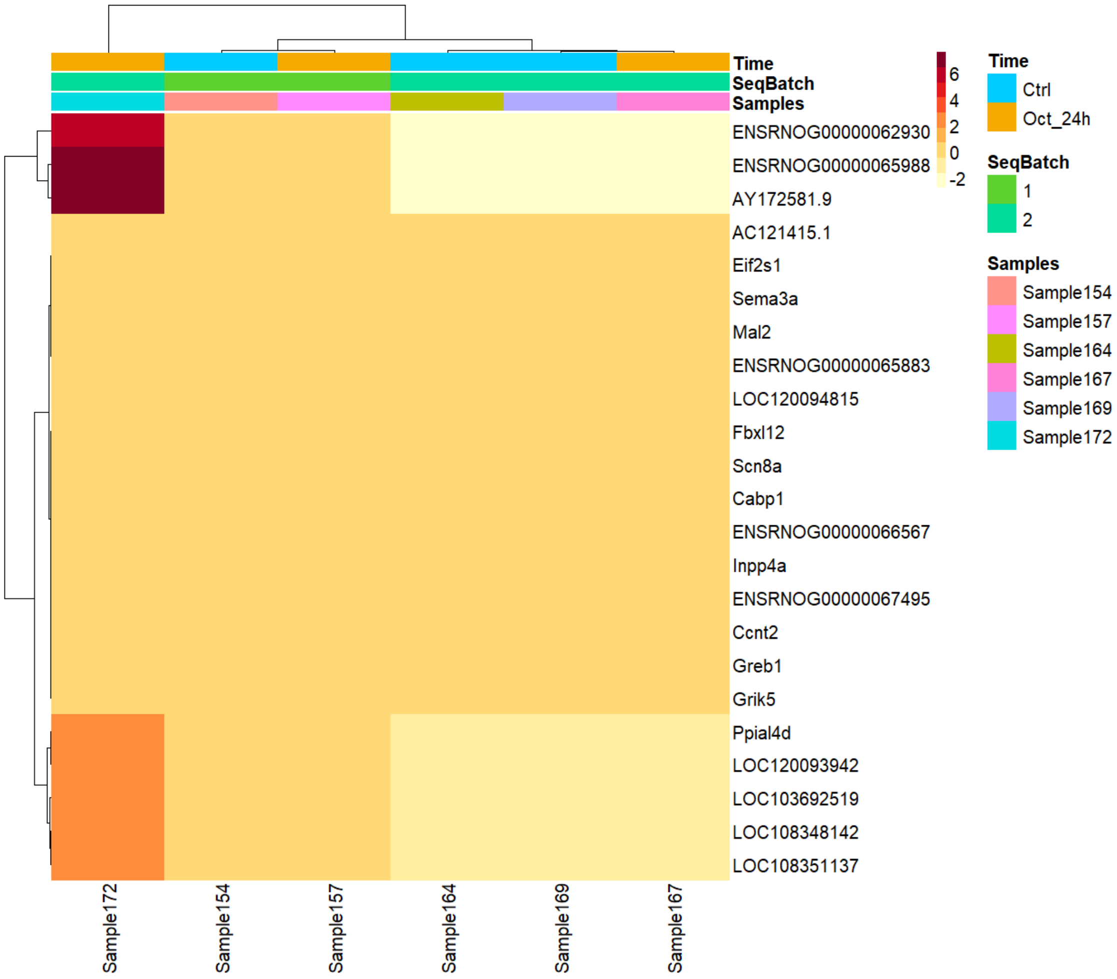

Figure S19. Variance stabilization transformed heatmap of mean normalized gene level counts for DEGs of the octreotide incubated GH3 cell and GH3 control comparsion at 24 hours. Light blue fields in the "Time" column row, represent the unincubated control samples, orange fields represent the GH3 samples incubated with octreotide at 24 hours.

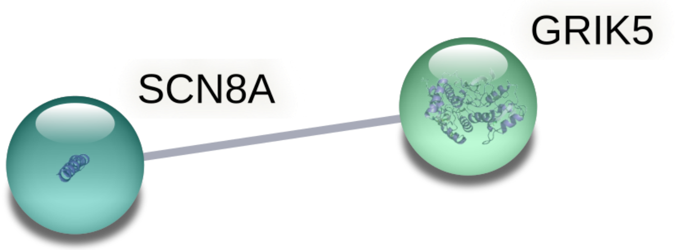

Figure S20. A STRING-db network for the DEG proteins of octreotide incubated GH3 cell and GH3 control comparsion for the incubation period of 24 hours. Line thickness indicates interaction confidence. Disconnected nodes were hidden. Text mining disabled as interaction type.

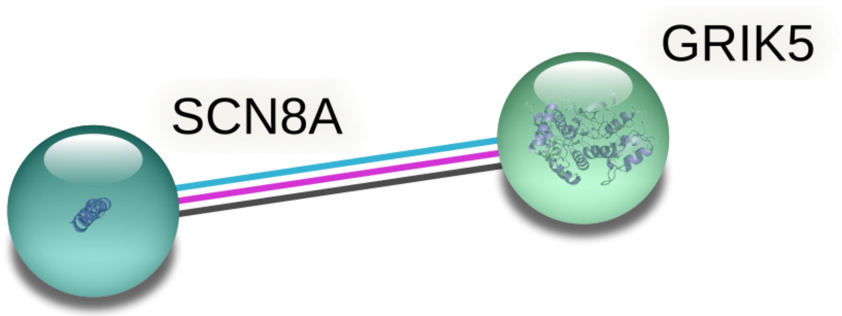

Figure S21. A STRING-db network for the DEG proteins of octreotide incubated GH3 cell and GH3 control comparsion for the incubation period of 24 hours. Line colour indicated interaction type and line count indicates number of interactions. Disconnected nodes were hidden. Text mining disabled as interaction type.
